# Supplementary material for: Mixed-methods study in England and Northern Ireland to understand young men who have sex with men’s knowledge and attitudes towards human papillomavirus vaccination
Source: BMJ Open. 2019 May 14;9(5):e025070. doi: 10.1136/bmjopen-2018-025070 (PMC6530382; doi:10.1136/bmjopen-2018-025070)
Supplement: Supplementary data [file bmjopen-2018-025070supp001.pdf]

Supplementary material A. Young HIMMS men-who-have-sex-with-men questionnaire

Demographic Information

1. What is your post code: ___________

2. What age are you: ___________

3. Are you:
   - ☐ Male (including trans)
   - ☐ Female (including trans)
   - ☐ Other (Non-Binary, Gender Neutral etc. Please specify): ____________

4. Do you identify as the following:
   - ☐ Gay Man
   - ☐ Bisexual
   - ☐ Man who has sex with men
   - ☐ Heterosexual
   - ☐ Unsure/Other (Please specify): ____________

5. Have you ever in the past had sex with another man or do you plan to in the future?
   - ☐ Yes
   - ☐ No
   - ☐ Unsure

   IF YOU ANSWER 'NO' TO THE ABOVE QUESTION YOU DO NOT NEED TO COMPLETE ANY FURTHER QUESTIONS.

6. Present cigarette smoking status
   - ☐ Non Smoker
   - ☐ Smoker
   - ☐ Former

7. To which ethnic group do you consider yourself to belong to?
   - ☐ Bangladeshi
   - ☐ Black African
   - ☐ Black Caribbean
   - ☐ Central European
   - ☐ Eastern European
   - ☐ Indian
   - ☐ Pakistani
   - ☐ Chinese
   - ☐ White
   - ☐ Mixed ethnicity
   - ☐ Other (please specify) _________________

8. What is your current relationship status?
   - ☐ Single

- ☐ Married
- ☐ Divorced
- ☐ Civil Partnership
- ☐ Dissolved partnership
- ☐ Co-habiting
- ☐ Separated
- ☐ In a relationship

9. Occupation/profession:

- ☐ Presently working full time
- ☐ Presently working part time
- ☐ Unemployed
- ☐ Student
- ☐ Training/apprentice

10. Indicate ALL people who live with you:

- ☐ No-one; I live alone
- ☐ Spouse/partner
- ☐ Parents
- ☐ Siblings
- ☐ Lives with others (not partner)
- ☐ Other (please specify) ___________________________

11. What is your religious preference?

- ☐ Non-religious
- ☐ Baptist
- ☐ Brethren
- ☐ Buddhist
- ☐ Catholic
- ☐ Church of Ireland
- ☐ Methodist
- ☐ Presbyterian
- ☐ Sikh
- ☐ Church of England
- ☐ Church of Scotland
- ☐ Free Presbyterian
- ☐ Hindu
- ☐ Jewish
- ☐ Muslim
- ☐ Protestant
- ☐ Other (please specify) ____________________________

Sexual Contact

12. Are you sexually active [for example sexual intercourse, oral sex, mutual masturbation]
    - ☐ Yes
    - ☐ No

IF YOU ANSWERED 'YES' TO QUESTION 12, PLEASE ANSWER QUESTIONS 13 – 18. IF YOU ANSWERED 'NO' PLEASE SKIP TO QUESTION 19.

13. How many male sexual partners have you had in the past 12 months:___________

14. What type of intercourse have you had in the past 12 months:
    - ☐ Anal intercourse only
    - ☐ Oral intercourse only
    - ☐ Both oral and anal intercourse

15. In the past 12 months have you used condoms (including oral sex):
    - ☐ Always
    - ☐ Sometimes
    - ☐ Never
    - ☐ Rather not say

16. Do you access sexual health services?
    - ☐Yes
    - ☐ No

    If 'No', can you give a reason as to why you don't? __________________________

    If 'Yes', which setting do you use for sexual health?
    - ☐ GUM
    - ☐ GP
    - ☐ LGBT service provider
    - ☐ non-LGBT service provider
    - ☐ HIV Clinic
    - ☐ Other (please specify): ___________

17. Have you ever been diagnosed with a sexually transmitted infection (STI)?
    - ☐ Yes
    - ☐ No
    - ☐ Rather not say

      If yes, please provide details: ___________________________________

18. Have you ever been diagnosed/treated for genital warts?
    - ☐ Yes
    - ☐ No
    - ☐ Rather not say

19. Have you been diagnosed with HIV
    - ☐ Yes

☐ No
☐ Rather not say

20. Where is the first contact you usually have with people you have sex with?
☐ Bars/Clubs
☐ Saunas
☐ Websites used for sexual encounters e.g. Grindr, Gaydar, Scruff
☐ Facebook/Twitter
☐ Dating websites
☐ Through friends
☐ Other (please specify)
____

Culture

21. Is your doctor aware of your sexual orientation?
☐ Yes
☐ No
☐ Not sure

22. Would you tell a health care professional that you have sex with other men or are interested in having sex with other men in order to receive a HPV vaccination?
☐ Yes
☐ No
☐ Not sure

23. If yes, what age would you feel comfortable disclosing you had sex with men in order to receive the HPV vaccine? _______________________

24. Have you talked to your doctor or other health care professional about HPV vaccination?
☐ Yes
☐ No
☐ Rather not say

If yes, please provide details

____

25. Has a health care professional ever recommended the HPV vaccine to you?
☐ Yes
☐ No
☐ Not sure

If yes, please provide details

____

26. If you were interested in receiving the HPV vaccine, where would you feel most comfortable receiving it?

☐ GUM
☐ GP surgery
☐ LGBT service provider
☐ non-LGBT service provider
☐ HIV Clinic
☐ Other (please specify)
____

27. The government is considering offering the HPV vaccine to men who have sex with men at Gum/HIV Clinics/GP surgeries. What are your views on this?

____

____

____

HPV and HPV VACCINE KNOWLEDGE

Have you heard about the human papillomavirus vaccine before today?

YES ☐

NO ☐

IF YOU HAVE ANSWERED 'NO' YOU DO NOT NEED TO COMPLETE ANY FURTHER QUESTIONS IN THIS SURVEY

IF 'YES' PLEASE READ THE FOLLOWING STATEMENT AND TICK THE APPROPRIATE RESPONSE FOR YOU:

The JCVI has recommended men who have sex with men aged up to 45 years receive the HPV vaccine.

Which of the following best describes your thoughts on the HPV vaccine for **men-who-have-sex-with-men**?

I have never thought about vaccination against HPV	☐
I am undecided about vaccination against HPV	☐
I have decided and do not want to vaccinate myself against HPV	☐
I have decided to do want to vaccinate myself against HPV	☐
I have already been vaccinated against HPV	☐

Can you time how long it takes you to complete this next section of the survey?

Please answer the following questions to the best of your ability:

	True	False	Don't know
1. *HPV is very rare*			
2. *HPV always has visible signs or symptoms*			
3. *HPV can be transmitted through genital skin-to-skin contact*			
4. *There are many types of HPV*			
5. *HPV can cause HIV/AIDS*			
6. *HPV can cause genital warts*			
7. *Men cannot get HPV*			
8. *Using condoms reduces the chances of HPV transmission*			
9. *HPV can be cured with antibiotics*			
10. *Having many sexual partners increases the risk of getting HPV*			
11. *HPV usually doesn't need any treatment*			
12. *Most sexually active people will get HPV at some point in their lives*			
13. *Having sex at an early age increases the risk of getting HPV*			
14. *HPV can cause cancer in men*			
15. *HPV is a bacterial infection*			
16. *HPV can be transmitted through oral sex*			
17. *HPV can cause herpes*			
18. *HPV can be transmitted through anal sex*			
19. *HPV infections always lead to health problems*			
20. *A person with no symptoms cannot transmit the HPV infection*			
21. *The HPV vaccines offer protection against all sexually transmitted infections*			
22. *The HPV vaccines are most effective if given to people who've never had sex*			

23. *One of the HPV vaccines offers protection against genital warts*			
24. *The HPV vaccine protects you from every type of HPV*			
25. *You can cure HPV by getting the HPV vaccine*			

Time for completion (in minutes): _________________________

HPV AND HPV VACCINE ATTITUDE

Can you time how long it takes you to complete this next section of the survey?

For each statement, please indicate how much you disagree or agree by selecting the appropriate number :							
	Strongly Disagree 1	***Disagree 2***	***Somewhat Disagree 3***	***Neutral 4***	***Somewhat Agree 5***	***Agree 6***	***Strongly Agree 7***
1. *I feel that the HPV vaccine will protect my sexual health.*							
2. *I feel that the HPV vaccine is effective in preventing HPV.*							
3. *I feel that the HPV vaccine is effective in preventing genital warts.*							
4. *I feel that the HPV vaccine is effective in preventing HPV-related cancers.*							
5. *I feel that vaccinating against HPV would protect my current/future partner from getting infected with HPV.*							
6. *I feel that it would be serious if I contracted HPV.*							
7. *I feel that it would be serious if I contracted genital warts.*							
8. *I feel that it would be serious if I contracted an HPV-related cancer.*							
9. *I feel that doctors/health care providers believe vaccinating* ***men-who-have-sex-with-men*** *against HPV is a good idea.*							
10. *I feel that the government believes I should vaccinate myself against HPV.*							
11. *The opinion of doctors/health care providers about getting the HPV vaccine matters to me.*							
12. *I trust the government's opinion concerning the HPV vaccine for* ***men-who-have-sex-with-men.***							
13. *I trust scientific evidence*							

concerning the HPV vaccine.							
14. *I have heard that the HPV vaccine is unsafe.*							
15. *I feel that the HPV vaccine might cause short term side-effects like pain or discomfort.*							
16. *I feel that the HPV vaccine is being pushed to make money for pharmaceutical companies.*							
17. *I feel that getting the HPV vaccine would be like performing an experiment on me.*							
18. *I feel that the HPV vaccine would encourage me to have sex with more partners.*							
19. *I feel that vaccinating for HPV would mean that I would not have to use safe sex practices.*							
20. *I feel that the HPV vaccine may lead to long-term health problems.*							
21. *I feel that the HPV vaccine may affect my fertility.*							
22. *I feel that getting the HPV vaccine would take too much effort.*							
23. *I feel that it is hard to find a clinic that would be easy to access for getting the HPV vaccine.*							
24. *I feel that I do not have enough information about the HPV vaccine.*							
25. *I feel that there has not been enough research done on the HPV vaccine.*							
26. *I feel that I am uncomfortable discussing my sexual health with a doctor/health care provider.*							
27. *I feel that I am uncomfortable talking about the HPV vaccine.*							
28. *I feel that the process of actually getting the HPV vaccine would be easy.*							

29. I feel that the HPV vaccine requires too many doses.							
30. I feel that vaccines are a good way to protect public health.							
31. I do not like the idea of vaccines.							
32. I feel that doctors give out too many vaccines.							
33. I feel that 16 is too young to receive the HPV vaccine.							

Time for completion (in minutes): _________________________
