# Supplementary material for: Mixed-methods study in England and Northern Ireland to understand young men who have sex with men’s knowledge and attitudes towards human papillomavirus vaccination
Source: BMJ Open. 2019 May 14;9(5):e025070. doi: 10.1136/bmjopen-2018-025070 (PMC6530382; doi:10.1136/bmjopen-2018-025070)
Supplement: Supplementary data [file bmjopen-2018-025070supp002.pdf]

Supplementary material B. Young MSM interview/focus group topic guide

1. Introduction *(5-10 min)*

Explain purpose of focus group

Before we begin I'm going to give some background to the study, an overview of the study's aim, the purpose of this focus group and details of who is funding the work.

In this focus group/interview we are going to be discussing Human papillomavirus (HPV) which is a very common infection involved in most cervical cancers. It is transmitted via skin-to-skin contact, most commonly during sexual activity. A vaccine has been developed that protects against this infection (A.S. Forster et al. / Vaccine 30 (2012) 4505–4510). It has recently been recommended that men who have sex with men (MSM) are offered this vaccination.

There are two purposes of this study. Firstly we want to understand the knowledge and attitudes of young (16-24 years) MSM towards the HPV vaccination. Secondly we are trying to identify ways to support young MSM to have this vaccination. We are particularly interested in how to support young MSM as the HPV vaccination is expected to provide greatest protection if it is given before the first sexual encounter.

The aim of this interview/focus group is to explore your knowledge and attitudes towards this vaccine, to identify things which may encourage or discourage vaccination and possible strategies to support vaccination uptake.

These interviews will contribute towards recommendations for any efforts to support the targeted vaccination of MSM, particularly those younger than 24 years of age.

This study has been funded by Cancer Research UK.

Explain audio recording procedures

Before we get started, I'd like to tell you that I will be recording the conversation to help us remember what we discussed and so that verbatim quotes can be used in future publications. You can ask for the recording to be stopped at any time and you can stop participating at any time without having to give a reason. What you say will be kept confidential and anonymous.

Guidelines for focus groups only

- Honesty – no right or wrong answers. Everyone's experiences and opinions are important. Feel free to agree or disagree with the views of others in the group.
- Confidentiality – We want people to feel comfortable about sharing potentially sensitive information so please do not discuss what is said during the group with others outside.
- Respect – you may not agree with what is said by others in the group but it is important to show respect to each other and to allow everyone a turn to express their opinions.
- Audio recording – Where possible please try to ensure that only one person is speaking at a time to aid the audio recording and transcription.

Ensure participant(s) has(ve) copy of participant information sheet

Answer any questions

Complete consent form(s) and check they have been completed correctly

Commence audio recording

2. Names and ice breaker for focus groups only *(2-5 min)*

For the benefit of the audio recorder can you start by saying your name and what you are currently studying/what your occupation is?

3. Sources of information and advice *(10 mins)*

To begin I'd like us to discuss where you got or would get information / advice about sexual health issues before engaging in any form of sexual activity with another man. This includes kissing, masturbation/hand jobs, oral sex and anal sex.

Where did you / would you receive or look for sexual health information and advice?

What are your reasons for choosing these places to find information or advice?

For which types of sexual activities are you most likely to seek advice?

Did you / would you consider speaking to a healthcare professional, including GPs, university health services or GUM clinics?

What are your reasons for doing / not doing this?

Could the information or advice you received or are currently receiving have been improved at all?

4. Perceptions of HPV risk *(5-10 mins)*

Now I'd like us to talk a little bit about your sexual health concerns in terms of Sexually Transmitted Diseases.

Firstly, what Sexually Transmitted Diseases do you know of?

I have a set of cards with Sexually Transmitted Diseases on (HPV, Hep A, Hep B, Hep C, Syphilis, HIV, Gonorrhoea, Genital Warts etc.) and I'd like you to order them in terms of what you are most to least concerned about as a group.

TAKE A PHOTO OF THE ORDER.

Can you talk me through your reasons for ordering the concerns like this?

5. Attitude towards HPV vaccination *(30 mins)*

I'd like us to move on now to discuss your views about being offered the HPV vaccination. The vaccine is most protective if it is received prior to first sexual encounter as this represents a potential exposure to HPV. Before being offered the vaccination it is likely that you would be asked to reveal your sexual orientation to a healthcare professional.

Thinking back to when you first disclosed your sexual orientation to someone, who did you disclose to?

Has any healthcare professional ever asked you about your sexual orientation?

If yes, did this happen before or after you had sex with another man?

If yes, how did the healthcare professional ask you for this information?

What were the circumstances in which the healthcare professional asked you for this information?

Could the way this information was asked have been improved at all? If yes, how?

If no, how happy would you be you to disclose your sexual orientation to a healthcare professional? What are your reasons for this?

Are there any types of healthcare professional that you would feel more comfortable disclosing your sexual orientation to than others (e.g. school nurse, GP, GUM clinic staff)?

What do you think is the best way for healthcare professionals to identify young (16-24 years) MSM who may be eligible for a HPV vaccine?

Prompts to be used if necessary:

- Through parents e.g. letters home to parents through school
- In private without parents/guardians
- Using a written questionnaire given in healthcare setting from 12/13 onwards?
- Face-to-face
- Via community LGBT organisations?

Who would you prefer to offer the HPV vaccination to you (e.g. GP, GUM clinic, school nurse etc.)?

Has anyone been offered or requested the HPV vaccine? (e.g. privately)

For what reason do you think you were offered/did you request the HPV vaccine?

How would you react to being offered the HPV vaccine?

How willing would you be to go and ask to have the HPV vaccine?

What things might prevent you or make you less likely to ask for/ accept the HPV vaccination?

What things might encourage you or make you more likely to ask for/accept the HPV vaccination?

6. Strategies to support the introduction of HPV vaccination in MSM *(30 mins)*

In the last set of questions we'd like to discuss your views on the best approach to encouraging the uptake of HPV vaccination in young MSM.

How could we increase young MSM awareness of the need to receive the HPV vaccination?

How could young MSM be encouraged to take up the HPV vaccine?

Prompts

- Awareness campaigns through schools, GUM clinics, social media etc.

7. Close *(2-3 mins)*

That is the end of my questions. Before we finish is there anything I haven't covered today that you would like to add?

We would like you to read our interpretation of the focus group. This shall be done by us sending you an email summary of the group discussion. We would like you to let us know if you feel it is an accurate interpretation of what was discussed. If you would like to do this, please provide us with an email address. This will not be kept confidential, and only used for this purpose.

End audio recording

Thank participant(s) and answer questions.
