# Supplementary material for: Mixed-methods study in England and Northern Ireland to understand young men who have sex with men’s knowledge and attitudes towards human papillomavirus vaccination
Source: BMJ Open. 2019 May 14;9(5):e025070. doi: 10.1136/bmjopen-2018-025070 (PMC6530382; doi:10.1136/bmjopen-2018-025070)
Supplement: Author's manuscript [file bmjopen-2018-025070.draft_revisions.pdf]

BMJ Open is committed to open peer review. As part of this commitment we make the peer review history of every article we publish publicly available.

When an article is published we post the peer reviewers' comments and the authors' responses online. We also post the versions of the paper that were used during peer review. These are the versions that the peer review comments apply to.

The versions of the paper that follow are the versions that were submitted during the peer review process. They are not the versions of record or the final published versions. They should not be cited or distributed as the published version of this manuscript.

BMJ Open is an open access journal and the full, final, typeset and author-corrected version of record of the manuscript is available on our site with no access controls, subscription charges or pay-per-view fees (http://bmjopen.bmj.com).

If you have any questions on BMJ Open's open peer review process please email info.bmjopen@bmj.com

BMJ Open

A mixed-methods study to understand young men-who-have-sex-with-men's knowledge and attitude towards Human Papillomavirus vaccination

Journal:	*BMJ Open*
Manuscript ID	bmjopen-2018-025070
Article Type:	Research
Date Submitted by the Author:	28-Jun-2018
Complete List of Authors:	Kesten, Joanna; NIHR Health Protection Research Unit in Evaluation of Interventions and NIHR CLAHRC West, University of Bristol, Bristol Medical School, Population Health Sciences<br>Flannagan, Carrie; Ulster University , Institute of Nursing and Health Research, School of Nursing<br>Ruane-McAteer , Eimear ; Queen's University Belfast, School of Nursing and Midwifery, Medical Biology Centre<br>Merriel, Samuel; University of Bristol, Centre for Academic Primary Care<br>Nadarzynski, Tom ; University of Southampton, Department of Psychology<br>Shapiro, Gilla; McGill University, Department of Psychology<br>Rosberger, Zeev; McGill University, Department of Psychology<br>Prue, G; Queen's University Belfast, School of Nursing and Midwifery, Medical Biology Centre
Keywords:	vaccination, HPV, sexual health, adolescent, attitudes

SCHOLARONE™
Manuscripts

A mixed-methods study to understand young men-who-have-sex-with-men's knowledge and attitude towards Human Papillomavirus vaccination

Author names

Joanna May Kesten PhD[a,b], Carrie Flannagan PhD[c], Eimear Ruane-McAteer PhD[d], Samuel W D Merriel MSc[e], Tom Nadarzynski PhD[f], Gilla K. Shapiro MPA/MPP[g], Zeev Rosberger, PhD[g], Gillian Prue PhD[d]

Author affiliations

[a] The National Institute for Health Research Health Protection Research Unit in Evaluation of Interventions, School of Social and Community Medicine, University of Bristol, UK

[b] The National Institute for Health Research Collaboration for Leadership in Applied Health Research and Care West (NIHR CLAHRC West) at University Hospitals Bristol NHS Foundation Trust, UK

[c] Institute of Nursing and Health Research, School of Nursing, Ulster University, Newtownabbey BT37 0QB c.flannagan@ulster.ac.uk

[d] School of Nursing and Midwifery, Medical Biology Centre, Queen's University Belfast, UK, BT9 7BL, g.prue@qub.ac.uk, eruanemcateer01@qub.ac.uk

[e] Centre for Academic Primary Care, University of Bristol, 39 Whatley Road Clifton Bristol BS8 2PS UK sam.merriel@bristol.ac.uk

[f] University of Southampton, Faculty of Health Sciences, Department of Psychology, t.nadarzynski@soton.ac.uk

[g] Department of Psychology, McGill University, 2001 McGill College Avenue, Montreal, Quebec, Canada, H3A 1G1, gilla.shapiro@mail.mcgill.ca; zeev.rosberger@mail.mcgill.ca

For peer review only - http://bmjopen.bmj.com/site/about/guidelines.xhtml

For peer review only

Corresponding author details

Joanna May Kesten

Email: Jo.Kesten@bristol.ac.uk

Telephone: 0117 33 10008

Address: NIHR CLAHRC West, 9th Floor, Whitefriars, Lewins Mead, Bristol BS1 2NT

Acknowledgements

This study was funded by an Innovation award (#22091) from Cancer Research UK/BUPA Foundation. JK is partly funded by National Institute for Health Research (NIHR) Collaboration for Leadership in Applied Health Research and Care West (CLAHRC West) at University Hospitals Bristol NHS Foundation Trust and NIHR Health Protection Research Unit in Evaluation of Interventions. SM is the recipient of an academic clinical fellowship from NIHR. GS was supported by the Vanier Canada Graduate Scholarship and Queen Elizabeth II Diamond Jubilee Scholarship programmes.

Word count: 3859

For peer review only - http://bmjopen.bmj.com/site/about/guidelines.xhtml

Abstract

Objectives: Men-who-have-sex-with-men (MSM) are at greater risk for HPV-associated cancers. Since 2016, MSM have been offered the HPV vaccination, which is most effective when received prior to sexual debut, at Genitourinary Medicine clinics in the United Kingdom. This study aimed to understand young MSM's (YMSM) knowledge and attitudes towards human papillomavirus (HPV) vaccination.

Design: Questionnaires assessed YMSM demographics, sexual behaviour, culture, knowledge and attitudes towards HPV vaccination, and stage of vaccine decision-making using the Precaution Adoption Process Model. Focus groups explored sexual health information sources, attitudes, barriers and facilitators to vaccination, and strategies to support vaccination uptake. Questionnaire data were analysed using descriptive statistics and focus group data were analysed thematically.

Setting: Focus groups were conducted within Lesbian Gay Bisexual Trans Queer (LGBTQ) organisational settings and a university student's union in England and Northern Ireland. Questionnaire's were also completed online.

Results: Seventeen YMSM (*M*=20.5 years) participated in four focus groups and 51 (*M*=21.1 years) completed questionnaires. Over half of YMSM were aware of HPV (54.9%), yet few (21.6%) had previously discussed vaccination with a Healthcare Professional (HCP). Thematic analyses found YMSM were willing to receive the HPV vaccine. Vaccination programmes requiring YMSM to request the vaccine, particularly prior to sexual orientation disclosure to family and friends, were viewed as unfeasible. Educational campaigns explaining vaccine benefits were indicated as a way to encourage uptake.

Conclusions: The findings from this study suggest that to effectively implement HPV vaccination for YMSM, this population requires clearer information and greater discussion with their HCP. Future research should implement and evaluate strategies to support HPV vaccination and address barriers to uptake in YMSM such as reluctance to disclose sexual orientation to HCP, particularly before sexual

For peer review only - http://bmjopen.bmj.com/site/about/guidelines.xhtml

encounters. Universal male vaccination may be the most feasible and equitable option to protect YMSM.

Strengths and limitations of this study

- This is the first study in the UK to explore YMSM's knowledge and attitudes toward HPV vaccination.
- Use of a theoretical model of behaviour change, facilitates clear conceptualisation of health behaviour change and YMSM's stage of HPV vaccine decision making.
- The qualitative component obtained a diverse range of views of YMSM in England and Northern Ireland.
- Survey findings should be interpreted with caution due to the sample size.

Keywords

HPV, sexual health, adolescent, attitudes, vaccination

For peer review only

Introduction

Human papillomavirus (HPV), the most common sexually transmitted infection (STI) worldwide [1], has serious health consequences for men and women. HPV is recognised as a causative agent in cervical cancer, and is associated with anogenital tumours, oropharyngeal cancers, and genital warts [2]. While boys and girls aged 12-13 years are vaccinated in school in Australia [3 4], the current United Kingdom (UK) strategy of vaccinating all girls aged 12-13 years does not protect young men-who-have-sex-with-men (YMSM) against HPV infection and related diseases [5] as they will not benefit from herd immunity [6].

The UK Joint Committee on Vaccination and Immunisation's (JCVI) statement on MSM HPV vaccination [7] recommended that vaccination programmes be extended to MSM aged up to 45 years via Genitourinary Medicine (GUM) clinics. Mathematical modelling suggests that for MSM aged 40 or over, HPV vaccination in GUM clinics is likely to be an effective and cost-effective method of reducing HPV-related disease burden in MSM in England [8] and elsewhere. Northern Ireland, Scotland and Wales are offering the HPV vaccine to MSM attending GUM clinics. Following a pilot programme in England which found moderate uptake and did not report completion rates, vaccination is now offered in GUM clinics. The JCVI are also considering whether to extend the adolescent female vaccination programme to all males. An interim statement (July 2017) suggested that given the current high uptake in females, extending immunisation to all adolescent males is "highly unlikely to be cost effective in the UK" (p13) [9], although in November 2017, the final decision was delayed, and it is understood that further modelling is underway [10].

The absence of a vaccination programme for all adolescent boys leaves UK YMSM without funded access to the HPV vaccine at the recommended age (12-13 years) - before exposure to HPV[11]. There is often a delay between the age of first sexual contact with another man and disclosure of sexual orientation to a healthcare professional (HCP)[12], and it is likely that MSM will have multiple sexual

For peer review only - http://bmjopen.bmj.com/site/about/guidelines.xhtml

partners, resulting in increased risk of HPV acquisition before attending a GUM clinic [13]. The benefit of a targeted programme may be limited due to the likelihood that many MSM acquire HPV in their teenage years [13]. As not all MSM access GUM clinics, this setting may lead to insufficient vaccination coverage. Modelling work in Australia indicated that a HPV vaccination programme targeting boys only with at least 84% coverage would reduce HPV infection in MSM by 90% [14].

A systematic review found that MSM HPV vaccine knowledge was low and MSM did not consider themselves at risk of infection, although over half would accept the vaccine if offered [11]. Most of these studies were conducted in North America (and none in the UK), with MSM over 26 years. Minimal attention has been given to the knowledge and attitudes toward HPV vaccination among adolescent and YMSM (aged 16-24 years). This is an important area for research because MSM may acquire HPV at a young age, close to their sexual debut (the age of which is decreasing) [13]. This study aimed to examine the knowledge and attitudes of UK YMSM towards HPV vaccination to inform policy and practice recommendations for accessing this hard to reach group, supporting vaccination uptake and the optimisation of protection from HPV.

Methods

Study design

We conducted questionnaires and focus groups with YMSM aged 16-24 years.

Participant eligibility and recruitment

The survey was administered online and advertised via various LGBTQ organisations. In addition, prior to the focus groups, participants were asked to complete the questionnaire. We have combined both the online and pen and paper completions for this paper.

For peer review only - http://bmjopen.bmj.com/site/about/guidelines.xhtml

We aimed to achieve data saturation [22] by recruiting 8-10 YMSM per focus group with a mix of social background, age, ethnicity, and religion. YMSM was defined through self-identification as male (including transgender male), at or over the age of sexual consent, sexually attracted to men, or had sex with a man [13]. Age inclusion criteria were based on the World Health Organisation's definition of "young": 15 – 24 years. A minimum of 16 years was specified as it is the age of sexual consent in the UK.

For the focus groups, potential participants were provided with written study information, and asked to register their interest at local Lesbian Gay Bisexual Transgender Queer (LGBTQ) advocacy groups, university information days, university student union clubs and societies, and secondary school LGBTQ groups. Advocacy groups advertised the study through social media and snowball sampling was employed.

Data collection

CF conducted the focus groups within LGBTQ organisational settings and a university student's union building.

The questionnaires (Supplementary Material A) assessed demographics (adapted from Hickson *et al.*)[15]; sexual behaviour (adapted from Sadlier *et al.*)[16]; culture (adapted from Zou *et al.*) [17]; and HPV vaccine stage of decision making using the Precaution Adoption Process Model (PAPM) [18]. The PAPM has six stages of behaviour change decision-making and has been used to examine knowledge and attitudes to HPV vaccination[19]. Those who indicated awareness of the HPV vaccine, were asked to complete validated HPV knowledge/attitudes scales [20 21].

The focus group topic guide (Supplementary Material B) was applied flexibly to allow for emergent issues and began by exploring sources of sexual health information and advice before engaging in sexual activity (not presented here). Perceptions of HPV risk in relation to six other STI's were then discussed using a sorting task in which a list of STIs were ordered by what is least to most concerning

For peer review only - http://bmjopen.bmj.com/site/about/guidelines.xhtml

(findings not reported here). Attitudes towards HPV vaccine, barriers and facilitators to vaccination and possible intervention strategies to support vaccination uptake were explored. Experiences of disclosing sexual orientation to HCP were also discussed. All participants were informed that the HPV vaccine was most protective if received prior to first sexual encounter. Participants were asked to reflect as to how they would have viewed taking the vaccine when they were 12-13 years.

Patient and Public Involvement

The HPV knowledge/attitude questionnaire scales were adapted for use with MSM through consultation with an Expert Panel including a key stakeholder group (The Rainbow Project [TRP]) and MSM focus groups.

YMSM were not involved in the development of the qualitative component of this study, however staff from the TRP helped develop the study design and documentation.

Analysis

Participants' PAPM vaccine decision-making stage was classified into six stages: unaware, unengaged, undecided, decided not to vaccinate, decided to vaccinate and those who had already been vaccinated [19]. Knowledge and attitudes held by participants about HPV and HPV vaccination were analysed using descriptive statistics.

Focus groups were audio recorded, transcribed verbatim, anonymised and analysed thematically [23] using QSR NVivo (version 10.0). This approach was chosen because it offers a clear analysis process while remaining flexible [23]. JK and CF independently coded the first transcript systematically, line-by-line, compared their coding and reached consensus. These initial codes were then applied to the remaining transcripts. The content of all the codes was read and compared to each other to iteratively

For peer review only - http://bmjopen.bmj.com/site/about/guidelines.xhtml

refine and cluster codes into themes and sub-themes. A description of each theme capturing instances of divergence was then written.

Ethical approval was granted from the Queens University Belfast, School of Nursing and Midwifery Research Ethics Committee (39.GPrue.05.16.M8.V2). Written informed consent from each participant was obtained prior to participation.

Results

Participant characteristics

Between September 2016 and March 2018, questionnaires were completed by 51 YMSM. Four focus groups in Northern Ireland (*n*=3) and England (*n*=1) were conducted between September and December 2016 with 17 YMSM (Table 1). Focus group size ranged from 2-6 participants and lasted a mean of 44.34 minutes (range=40.4-50.4).

Table 1. Participant characteristics

Questionnaire results

The majority (*n*=49) were sexually active and reported both oral and anal intercourse in the past 12 months (*n*=35), a wide range of partner numbers (*M*= 5 partners, range 0-25), and 'sometimes' (*n*=17) or 'never' (*n*=16) used condoms. 29 participants had accessed sexual health services (Table 2).

For peer review only

Nineteen participants (37%) had never heard of HPV. Of those who had heard of HPV in accordance with the PAPM, 18% were in the 'decided to act' stage of vaccine decision-making (stage 5), none had decided that they did not want the vaccine (stage 4), and 22% had already been vaccinated (stage 6) (Table 3).

Of those who were aware of HPV (*n*= 28), knowledge of HPV, and the HPV vaccine was generally high; mean items correct 65% (*M*= 13.3, SD 4.7) and 60% (*M*= 3.3, SD, 1.2), respectively. However, there was wide variation in knowledge scores (HPV range, 3-20; HPV vaccine range, 0-5) (Table 3). Participants were aware that HPV affected men, the method of HPV transmission and that vaccination was most effective if given prior to sexual debut; however, awareness of the link between HPV and genital warts and the severity of an HPV infection was lower as the majority of YMSM thought HPV infection always required treatment and that infection with HPV would always lead to health problems (Table 3).

Thirty-three participants reported that HPV vaccination had never been discussed with or recommended by a HCP (Table 3). The mean age participants were willing to disclose their sexuality to a HCP was 18.3 years (range=11-23, *SD*=2.40) (Table 3). The most comfortable setting cited to receive the HPV vaccine was primary care or LGBTQ-specific services, rather than GUM clinics (Table 3).

Table 2. Sexual contact and relationships

Table 3. HPV vaccine: culture, awareness and stage of decision making

For peer review only - http://bmjopen.bmj.com/site/about/guidelines.xhtml

Qualitative results

Anonymous quotes illustrating two key themes are presented below.

1. Willingness to be vaccinated

Despite a perceived lack of knowledge about HPV and the vaccine and the threat posed to men, most participants were willing to receive the vaccine and wanted more information.

P1: I only knew about it because of the cervical cancer (…)

P2: I didn't even know that was what it was for.

P1: I didn't know even if like that would apply to us, so I don't even know what the dangers are.

Focus group 2

Participants were motivated to receive the vaccine to protect their health and a small number of participants suggested that the cost and number of doses of the vaccine were not barriers to vaccination.

I'm not going to say like get rid of worry because you still have to…it's your sexual health, but it would be safer in a sense (…) I'm better protected – I think would be a better way of putting it. So, I think my own health would encourage me more [to ask or accept the HPV vaccine]. I'd rather be protected than not protected.

Focus group 3, unidentifiable speaker

For peer review only - http://bmjopen.bmj.com/site/about/guidelines.xhtml

2. Implementation recommendations

Promoting and raising awareness of the vaccine

Better understanding of the benefits and side-effects of the vaccine were expected to encourage uptake. To promote the vaccine and inform YMSM, awareness campaigns and advertisements on the internet, radio, TV, social media, in University society's, LGBTQ youth groups and dating apps were suggested.

> *For this generation particularly, social media and TV ads and newspapers – well, probably not newspapers, but radio ads as well. You know, a campaign around getting people vaccinated, I think that would be very beneficial for young people these days.*
>
> Focus group 3, unidentifiable speaker

Participants suggested including information about the vaccine for YMSM in primary care and the sexual health education curriculum in schools. Indeed, it was noted that there is a lack of MSM-specific sexual health and relationship information provided in the latter.

> *When you're receiving that [heterosexual relationship education] in school, (…) it just reinforces the fact that you're (…) not relating to it means that you're not normal like everyone else, so you don't want to speak about it. So it would just be better if it [HPV vaccine education for MSM] was just part of that education anyway.*
>
> Focus Group 2

For peer review only - http://bmjopen.bmj.com/site/about/guidelines.xhtml

Identifying and offering YMSM the HPV vaccination

The ideal pre-exposure timing for vaccination and the fluid, undefined nature of sexual preferences at a young age were perceived as barriers to identifying eligible recipients. There were mixed feelings about whether it would be acceptable for HCPs to ask boys (<16 years) to disclose their sexuality for this purpose due to concern about parents being informed and a lack of a trusting relationship. It was, however, also noted that questions about sexuality need to be normalised, particularly in primary care.

> *Interviewer: If everybody was getting the HPV vaccine…*
>
> *Participant 2: That's probably what they should do, because, I mean, (...) someone might think now, oh, I'll never have sex with a man, but then, later down the line, they might do.*
>
> Focus group 4

The focus group participants wanted the benefits of vaccination to be explained and for the vaccine to be offered in a natural, relaxed manner, opportunistically, rather than having to request it. Participants felt they would be unlikely to request the vaccine because they would need more knowledge and they felt too uncomfortable.

> *Participant 2: As long as there was someone professional telling me what's it about, what's it going to do, and what it could do if it goes wrong.*
>
> *Focus group 2*

Participants reckoned it was not feasible to expect young boys to identify themselves for the HPV vaccine when they potentially had not disclosed or decided their sexual orientation. There was also a

For peer review only

preference for not singling boys out by their sexuality when offering the vaccine. Similarly, receiving the vaccine confidentially was important because the potential for bullying and embarrassment would act as barriers. It was noted by participants that universal vaccination of all boys would avoid these problems. A young person seeking sexual health advice represented an opportunity to identify eligible boys. However, this is likely to occur post-sexual encounter – after the risk of exposure to the virus.

I would want them to approach me. I wouldn't go out of my way to go and get it.

Focus group 3, unidentifiable speaker

Interviewer: So then you're asking Year 8 and 9 that age group (…) -

Unidentifiable participant: To basically out themselves…

[Agreement]

Interviewer: Do you see that as being a feasible scenario?

Unidentifiable participant: No.

Unidentifiable participant: Absolutely not.

Unidentifiable participant: The only kind of way round that is if every like male child is also vaccinated, but (…) obviously they won't do that because in terms of cost of vaccines.

Focus group 3, unidentifiable speaker

Participant 2: When you get your vaccinations in school, you all, (…) used to go in to get your vaccinations [as a class]. If it were like that, I wouldn't do it, because I wouldn't like anyone seeing.

For peer review only - http://bmjopen.bmj.com/site/about/guidelines.xhtml

Focus group 4

Participant 6: Why wouldn't it be offered to like young males in school, (...) so it was like before like presumably anybody had had sex (...). A lot more people would get it that way.

Focus group 1

There were mixed feelings about General Practitioners (GPs) or specialist sexual HCPs offering the vaccine. The relationship with the HCP was important; if YMSM have a good relationship with their GP then being offered the HPV vaccine by them is preferable. In contrast, a small number would feel more comfortable being offered the vaccine by someone they trust from a community LGBTQ group or local sexual health centre. A comment was also made about the nature of the vaccine being related to sexual health meaning it made more sense/was easier to offer it via specialist services. However, prior to disclosure or sexual activity, the participants commented that boys may not engage with or know about sexual health or LGBTQ services so offering the vaccine in these setting may represent a barrier.

Telling your family GP you're gay before you've told your family would be a big no I think because the GP might go back and tell your parents and then out you.

Focus group 3, unidentifiable speaker

If you have to go and ask about it and ask for it, who would you ask because you wouldn't be able to come here [Community LGBTQ group] because you wouldn't know here existed.

Focus group 4

For peer review only - http://bmjopen.bmj.com/site/about/guidelines.xhtml

Written invitations from GPs offering the vaccine to eligible patients were also suggested. However, this would require boys to identify as MSM when registering or being asked about their sexuality by a HCP. A small number of accounts suggested it would be acceptable to refer patients to receive the HPV vaccine in sexual health clinics if it was not available in a GP setting. Offering the vaccine in schools when YMSM are beginning to have their first sexual encounters was suggested. Similarly, the school nurse was a trusted individual for some and therefore may be an acceptable person to provide the vaccine.

Discussion

This is the first study to examine the views of YMSM towards the HPV vaccine in the UK. Despite being sexually active and willing to disclose sexual orientation to receive the vaccine, most participants had never been recommended the HPV vaccine, suggesting that MSM are not being offered the vaccine at the most opportune time. The data also suggested that HPV knowledge in YMSM is low, with almost half of participants being unaware of HPV or the vaccine. YMSM were willing to receive the vaccine but wanted additional information about HPV and the vaccine. Given the reluctance to disclose information about sexuality to HCP (prior to disclosure to significant others), the wide range of sexual partner numbers, and lack of consistent contraceptive use, combined with the importance of supporting vaccination prior to potential exposure, the findings highlight significant barriers to MSM accessing the vaccine. Early provision of information was recommended through awareness campaigns, advertisements and the school health education curriculum. However, even with enhanced awareness, programmes that rely on YMSM to present for vaccination (particularly prior to sexual orientation disclosure) were not viewed as feasible. Furthermore, preferences for GPs or specialist HCPs offering the vaccine were dependent on the relationship with the HCP. Offering the vaccine to MSM in schools was thought to be acceptable.

For peer review only - http://bmjopen.bmj.com/site/about/guidelines.xhtml

Strengths and Limitations

This is the first study in the UK exploring this topic with YMSM. By conducting this research in more than one setting we can comment on the transferability of our findings; we found minimal differences in attitudes towards HPV between settings. The use of a theoretical model of behaviour change, the PAPM, also facilitates clear conceptualisation of health behaviour change and YMSM's stage of HPV vaccine decision making.

We aimed to continue data collection until saturation, however recruitment difficulties and the study timeframe meant that the decision to cease recruitment was pragmatic. The sensitivity of the topic, the hard to reach population and the lack of monetary compensation for the participant's time are possible explanations for this. The sample size is however, considered appropriate as it has obtained a diverse range of views of YMSM. Those who self-selected to participate may be more comfortable with their sexuality than those who did not agree. Indeed, recruiting through LGBTQ organisations narrowed our participant pool to those engaged with these services who had disclosed their sexual orientation. The small sample size for the quantitative data resulted in a lack of statistical power and should be interpreted with caution. The interview sample age range of 16-24 years is older than the target population for the vaccine - 12-13 years. Although the participants were asked to consider how they would view the vaccine and strategies to implement it among YMSM, it is unclear whether current YMSM share similar attitudes.

Implications for research and practice

The reluctance of YMSM to discuss their sexuality with HCPs before they have disclosed to significant others has important implications for the success of an HPV vaccination programme. Previous research shows that MSM disclosing their sexuality to significant others, visiting HCPs in the past year, and previous STI diagnosis predict disclosure to a HCP [24]. In the absence of a universal

For peer review only - http://bmjopen.bmj.com/site/about/guidelines.xhtml

vaccination strategy for boys and girls, additional measures to support YMSM to access the vaccine are necessary. For instance, information may need to be provided to young men outside of healthcare settings including educational contexts during sex and relationship education or HCPs may need to take an active role in opportunistically providing information during consultations for non-sexual health related matters. To support the latter, GPs and other HCPs may require additional education and training [25, 26].

Comparison to existing literature

A lack of knowledge does not appear to deter MSM willingness to be vaccinated [27]. However, MSM in this study and in others [27], desired more information. Poor knowledge of the HPV vaccine among YMSM has also been reported previously [11].

Other qualitative work with MSM has shown support for vaccinating all adolescent boys in school in part to protect against stigma arising from vaccination policies targeting MSM [28]. This would also remove the barrier of MSM having to request the vaccination, especially prior to sexual debut [26].

Our finding that MSM are unlikely to disclose sexual orientation to a HCP prior to sexual debut, has been reported elsewhere [12], suggesting that HPV vaccine programmes delivered by HCPs would be of "limited benefit" [12]. Participants in our study recommended the vaccine be offered by HCPs rather than expecting them to request it, however it is unclear whether initial reluctance to disclose sexuality would prevent vaccination uptake. The absence of a HCP's recommendation has previously been identified as a barrier to vaccination [29]. A new NHS England standard recommending "sexual orientation monitoring" whereby patients aged 16 and over are asked to disclose their sexual orientation at every face-to-face appointment may help to identify those eligible for vaccination [30].

For peer review only - http://bmjopen.bmj.com/site/about/guidelines.xhtml

Although this standard would not help identify those younger than 16 years who may benefit from the vaccine.

Previous research has found that most MSM have positive attitudes towards vaccinations against STIs and would be willing to receive the HPV vaccine [28]. However, individual and systemic barriers, such as access to sexual health clinics, disclosure of sexual orientation, concern about confidentiality or belief that HPV vaccine is not effective after sexual debut, may compromise the effectiveness of vaccination strategies [28]. Additionally, perceptions that HPV is relatively uncommon and harmless may lead to low desirability of the vaccine resulting in suboptimal coverage and therefore reduced cost-effectiveness [28].

In line with our findings, awareness raising strategies are vital to HPV vaccination programme success [31 32]. To raise awareness and motivate vaccine uptake, a public health campaign may be necessary [27]. When developing strategies for HPV vaccination programmes, stakeholders can learn from the introduction of vaccinations such as hepatitis B and should engage with the target population and co-ordinate between stakeholders to ensure consistent messages [31].

Conclusions

This study suggests that UK YMSM's are willing to receive the HPV vaccine. However, the UK's current HPV vaccine programme that relies on MSM to present for vaccination (particularly prior to sexual orientation disclosure) was not viewed as feasible. The importance of supporting vaccination prior to potential virus exposure combined with the reluctance to disclose information about sexual orientation means personal knowledge and awareness of the HPV vaccine is important therefore, early provision of information is recommended. Offering the vaccine in healthcare and education settings

For peer review only - http://bmjopen.bmj.com/site/about/guidelines.xhtml

may be acceptable, although the barriers to this channel of provision may mean that universal vaccination is the most feasible and equitable option.

Table 1. Participant characteristics

**Participant characteristics**	**Questionnaire participants**			**Focus group participants**		
	***M* (*SD*)**	**Range**	***N***	***M* (*SD*)**	**Range**	**N (% of sample)**
Age (years)	21.06 (2.6)	16-24		20.5 (2.73)	16-24	18[1] (100)
Location						
- Northern Ireland			36			13 (72.2)
- England			15			5 (27.8)
Education						
- Full-time education			26			11
- Employed full-time			17			4
- Employed part-time			5			1
- Unemployed			2			1
- Missing			1			1

For peer review only

For peer review only - http://bmjopen.bmj.com/site/about/guidelines.xhtml

Group size	
Focus group 1[1]	6
Focus group 2	2
Focus group 3	4
Focus group 4	5

[1]One participant completed the questionnaire and left before the focus group began due to time constraints

For peer review only

For peer review only - http://bmjopen.bmj.com/site/about/guidelines.xhtml

Table 2. Sexual contact and relationships

Sexual contact and relationships	*M* (*SD*)	Range	*N (%)*
Have you ever in the past had sex with a man or do you plan to in the future?			
- Yes			49 (96.08%)
- No			1 (1.96%)
- Missing			1 (1.96%)
Relationship status:			
- Single			26 (50.98%)
- In a relationship			21 (41.18%)
- Co-habiting			2 (3.92%)
- Civil Partnership			1 (1.96%)
- Missing			1 (1.96%)
Are you sexually active?			
- Yes			38 (74.51%)
- No			8 (15.69%)
- Missing			5 (9.8%)
How many male sexual partners have you had in the past 12 months?	5 (6)	0-25	
What type of intercourse have you had in the past 12 months?			
- Oral only			3 (5.88%)
- Anal only			2 (3.92%)
- Both oral and anal			35 (68.63%)
- Neither			3 (5.88%)

For peer review only - http://bmjopen.bmj.com/site/about/guidelines.xhtml

- Missing	8 (15.69%)
In the past 12 months have you used condoms?	
- Always	9 (17.65%)
- Sometimes	17 (33.33%)
- Never	16 (31.37%)
- Prefer not to say	1 (1.96%)
- Missing	8 (15.69%)
Do you access sexual health services?	
- Yes	29 (56.86%)
- No	14 (27.45%)
- Missing	8 (15.69%)

For peer review only

Table 3. HPV vaccine: culture, awareness and stage of decision making

	M (*SD*)	Range	*N*
GP aware of sexuality			
- Yes			22 (43.14%)
- No			17 (33.33%)
- Not sure			8 (15.69%)
- Missing			4 (7.84%)
Willing to disclose MSM status to HCP to receive HPV vaccine?			
- Yes			41 (80.39%)
- No			3 (5.88%)
- Not sure			3 (5.88%)
- Missing			4 (7.84%)
If yes, at what age?	18.3 (2.4)	11-23	
Has a HCP ever recommended an HPV vaccine to you?			
- Yes			11 (21.57%)
- No			33 (64.71%)
- Not sure			1 (1.96%)
- Missing			6 (11.76%)
Discussed HPV vaccination with HCP			
- Yes			10 (19.61%)
- No			34 (66.67%)
- Missing			7 (13.73%)

For peer review only - http://bmjopen.bmj.com/site/about/guidelines.xhtml

Most comfortable setting to receive HPV vaccine*some ticked more than one option*:	17 (33.33%)
- Genitourinary medicine (GUM)	30 (58.82%)
- Primary care	33 (64.71%)
- Lesbian Gay Bisexual	1 (1.96%)
Transgender organisations	2 (3.92%)
- Non-LGBT specific sexual health	1 (1.96%)
provider	
- HIV clinic	
Prior awareness of HPV	
- Yes	28 (54.9%)
- No	19 (37.25%)
- Missing	4 (7.84%)
PAPM (Stage of vaccine decision making)	
- *Stage 2 Unengaged*: I have never thought about vaccination against HPV	17 (33.33%)
- *Stage 3 Undecided*: I am undecided about vaccination against HPV	2 (3.92%)
- *Stage 4 Decided not to act*: I have decided and do not want to vaccinate myself against HPV	0
- *Stage 5 Decided to act*: I have	9 (17.65%)

For peer review only

	decided and I do want to vaccinate myself against HPV			
-	*Stage 6 Acted*: I have already been vaccinated against HPV			11 (21.57%)
-	Missing			12 (23.53%)
Knowledge scores				
-	HPV knowledge score (max 20)	13.3 (4.7)	3-20	27
-	HPV vaccination knowledge score (max 5)	3.3 (1.2)	0-5	27

For peer review only - http://bmjopen.bmj.com/site/about/guidelines.xhtml

Funding: Project funded by CRUK/BUPA Foundation Fund Innovation Grant (#22091).

Competing interests: None declared.

Contributors: JK drafted the manuscript and led the analysis of the qualitative data supported by CF. CF conducted the focus groups and questionnaires. GP, JK and SM conceived the research question. ZR's research team developed the original questionnaires and use of the PAPM in college males and parents of young children eligible for the HPV vaccine (prior to adaption for MSM). GP led the research team. ERM and GP conducted the analysis of the questionnaire data. All co-authors developed the research question, methodology and supported the management of the project. All authors have read, contributed to and approved the final manuscript.

Participant consent: written consent obtained.

Ethics approval: This study was approved by the Queens University Belfast, School of Nursing and Midwifery Research Ethics Committee (39.GPrue.05.16.M8.V2).

Data sharing statement: No additional data are available.

For peer review only

References

1. Forman D, de Martel C, Lacey CJ, et al. Global burden of human papillomavirus and related diseases. *Vaccine* 2012;30 Suppl 5:F12-23. doi: 10.1016/j.vaccine.2012.07.055 [published Online First: 2012/12/05]

2. Liddon N, Hood J, Wynn BA, et al. Acceptability of human papillomavirus vaccine for males: a review of the literature. *The Journal of adolescent health : official publication of the Society for Adolescent Medicine* 2010;46(2):113-23. doi: 10.1016/j.jadohealth.2009.11.199 [published Online First: 2010/02/02]

3. Prevention.; CfDCa. Human Papillomavirus (HPV). Genital HPV Infection - Fact Sheet 2017 [accessed 31st October 2017.

4. Health AGDo. Immunise Australia Program Human Papillomavirus (HPV) Australian Goverment Department of Health2017 [Available from: http://www.immunise.health.gov.au/internet/immunise/publishing.nsf/Content/immunise-hpv accessed 30th November 2017.

5. Read TR, Hocking JS, Chen MY, et al. The near disappearance of genital warts in young women 4 years after commencing a national human papillomavirus (HPV) vaccination programme. *Sexually transmitted infections* 2011;87(7):544-7. doi: 10.1136/sextrans-2011-050234 [published Online First: 2011/10/06]

6. Machalek DA, Poynten M, Jin F, et al. Anal human papillomavirus infection and associated neoplastic lesions in men who have sex with men: a systematic review and meta-analysis. *The Lancet Oncology* 2012;13(5):487-500. doi: 10.1016/s1470-2045(12)70080-3 [published Online First: 2012/03/27]

7. Health.; Do, England. PH. JCVI statement on HPV vaccination of men who have sex with men 2015 [Available from: https://www.gov.uk/government/publications/jcvi-statement-on-hpv-vaccination-of-men-who-have-sex-with-men accessed 7th February 2016.

8. Lin A, Ong KJ, Hobbelen P, et al. Impact and Cost-effectiveness of Selective Human Papillomavirus Vaccination of Men Who Have Sex With Men. *Clinical infectious diseases :*

For peer review only - http://bmjopen.bmj.com/site/about/guidelines.xhtml

an official publication of the Infectious Diseases Society of America 2017;64(5):580-88. doi: 10.1093/cid/ciw845 [published Online First: 2016/12/25]

9. Health.; Do, England. PH. JCVI statement: extending the HPV vaccination programme 2017 [Available from: https://www.gov.uk/government/publications/jcvi-statement-extending-the-hpv-vaccination-programme accessed 8th August 2017.

10. Immunisation JCoVa. Joint Committee on Vaccination and Immunisation [minute 2017 10.Draft.pdf]: Gov.UK; 2017 [Available from: https://www.gov.uk/government/groups/joint-committee-on-vaccination-and-immunisation#minutes.

11. Nadarzynski T, Smith H, Richardson D, et al. Human papillomavirus and vaccine-related perceptions among men who have sex with men: a systematic review. *Sexually transmitted infections* 2014;90(7):515-23. doi: 10.1136/sextrans-2013-051357 [published Online First: 2014/05/03]

12. Rank C, Gilbert M, Ogilvie G, et al. Acceptability of human papillomavirus vaccination and sexual experience prior to disclosure to health care providers among men who have sex with men in Vancouver, Canada: implications for targeted vaccination programs. *Vaccine* 2012;30(39):5755-60. doi: 10.1016/j.vaccine.2012.07.001 [published Online First: 2012/07/17]

13. Zou H, Tabrizi SN, Grulich AE, et al. Early acquisition of anogenital human papillomavirus among teenage men who have sex with men. *The Journal of infectious diseases* 2014;209(5):642-51. doi: 10.1093/infdis/jit626 [published Online First: 2013/11/23]

14. Zhang L, Regan DG, Ong JJ, et al. Targeted human papillomavirus vaccination for young men who have sex with men in Australia yields significant population benefits and is cost-effective. *Vaccine* 2017;35(37):4923-29. doi: 10.1016/j.vaccine.2017.07.078 [published Online First: 2017/08/10]

15. Hickson F, Tomlin K, Hargreaves J, et al. Internet-based cohort study of HIV testing over 1 year among men who have sex with men living in England and exposed to a social marketing intervention promoting testing. *Sexually transmitted infections* 2015;91(1):24-30. doi: 10.1136/sextrans-2014-051598 [published Online First: 2014/10/02]

For peer review only - http://bmjopen.bmj.com/site/about/guidelines.xhtml

16. Sadlier C, Rowley D, Morley D, et al. Prevalence of human papillomavirus in men who have sex with men in the era of an effective vaccine; a call to act. *HIV medicine* 2014;15(8):499-504. doi: 10.1111/hiv.12150 [published Online First: 2014/03/25]

17. Zou H, Grulich AE, Cornall AM, et al. How very young men who have sex with men view vaccination against human papillomavirus. *Vaccine* 2014;32(31):3936-41. doi: 10.1016/j.vaccine.2014.05.043 [published Online First: 2014/05/24]

18. Weinstein ND. The precaution adoption process. *Health psychology : official journal of the Division of Health Psychology, American Psychological Association* 1988;7(4):355-86. [published Online First: 1988/01/01]

19. Perez S, Tatar O, Shapiro GK, et al. Psychosocial determinants of parental human papillomavirus (HPV) vaccine decision-making for sons: Methodological challenges and initial results of a pan-Canadian longitudinal study. *BMC public health* 2016;16(1):1223. doi: 10.1186/s12889-016-3828-9 [published Online First: 2016/12/07]

20. Perez S, Shapiro GK, Tatar O, et al. Development and Validation of the Human Papillomavirus Attitudes and Beliefs Scale in a National Canadian Sample. *Sexually transmitted diseases* 2016;43(10):626-32. doi: 10.1097/olq.0000000000000506 [published Online First: 2016/09/16]

21. Perez S, Tatar O, Ostini R, et al. Extending and validating a human papillomavirus (HPV) knowledge measure in a national sample of Canadian parents of boys. *Preventive medicine* 2016;91:43-49. doi: 10.1016/j.ypmed.2016.07.017 [published Online First: 2016/07/30]

22. Glaser B, Strauss A. The discovery of grounded theory: Strategies for qualitative research. Chicago: Aldine 1967.

23. V.; B, V. C. Using thematic analysis in psychology. *Qual Res Psychol* 2006;3:77–101.

24. Stupiansky NW, Liau A, Rosenberger J, et al. Young Men's Disclosure of Same Sex Behaviors to Healthcare Providers and the Impact on Health: Results from a US National Sample of Young Men Who Have Sex with Men. *AIDS patient care and STDs* 2017;31(8):342-47. doi: 10.1089/apc.2017.0011 [published Online First: 2017/07/29]

For peer review only - http://bmjopen.bmj.com/site/about/guidelines.xhtml

25. Merriel S, Flannagan C, Kesten J, et al. Knowledge and Attitudes of General Practitioners and Sexual Health Care Professionals Regarding Human Papillomavirus Vaccination for Young Men Who Have Sex with Men. *International Journal of Environmental Research and Public Health* 2018;15(1):151.

26. Scully D, Irving N, Daly M, et al. HPV vaccination among men who have sex with men in Ireland: GPs' awareness and vaccination rates. *Sexually transmitted infections* 2017 doi: 10.1136/sextrans-2017-053372

27. Gutierrez B, Leung A, Jones KT, et al. Acceptability of the Human Papillomavirus Vaccine Among Urban Adolescent Males. *American Journal of Men's Health* 2012;7(1):27-36. doi: 10.1177/1557988312456697

28. Nadarzynski T, Smith H, Richardson D, et al. Perceptions of HPV and attitudes towards HPV vaccination amongst men who have sex with men: A qualitative analysis. *British Journal of Health Psychology* 2017;22(2):345-61. doi: 10.1111/bjhp.12233

29. Donahue KL, Stupiansky NW, Alexander AB, et al. Acceptability of the human papillomavirus vaccine and reasons for non-vaccination among parents of adolescent sons. *Vaccine* 2014;32(31):3883-5. doi: 10.1016/j.vaccine.2014.05.035 [published Online First: 2014/05/23]

30. Unit NEEaHI. Implementation Guidance Fundamental Standard for Sexual Orientation Monitoring 2017 [2017.

31. Sherris J, Friedman A, Wittet S, et al. Chapter 25: Education, training, and communication for HPV vaccines. *Vaccine* 2006;24 Suppl 3:S3/210-8. doi: 10.1016/j.vaccine.2006.05.124 [published Online First: 2006/09/05]

32. Brandt HM, McCree DH, Lindley LL, et al. An evaluation of printed HPV educational materials. *Cancer control : journal of the Moffitt Cancer Center* 2005;12 Suppl 2:103-6. [published Online First: 2005/12/06]

For peer review only - http://bmjopen.bmj.com/site/about/guidelines.xhtml

Supplementary material A. Young HIMMS men-who-have-sex-with-men questionnaire

Demographic Information

1. What is your post code: ____________

2. What age are you: ____________

3. Are you:
   - ☐ Male (including trans)
   - ☐ Female (including trans)
   - ☐ Other (Non-Binary, Gender Neutral etc. Please specify): _____________

4. Do you identify as the following:
   - ☐ Gay Man
   - ☐ Bisexual
   - ☐ Man who has sex with men
   - ☐ Heterosexual
   - ☐ Unsure/Other (Please specify): _____________

5. Have you ever in the past had sex with another man or do you plan to in the future?
   - ☐ Yes
   - ☐ No
   - ☐ Unsure

   IF YOU ANSWER 'NO' TO THE ABOVE QUESTION YOU DO NOT NEED TO COMPLETE ANY FURTHER QUESTIONS.

6. Present cigarette smoking status
   - ☐ Non Smoker
   - ☐ Smoker
   - ☐ Former

7. To which ethnic group do you consider yourself to belong to?
   - ☐ Bangladeshi
   - ☐ Black African
   - ☐ Black Caribbean
   - ☐ Central European
   - ☐ Eastern European
   - ☐ Indian
   - ☐ Pakistani
   - ☐ Chinese
   - ☐ White
   - ☐ Mixed ethnicity
   - ☐ Other (please specify) ___________________

8. What is your current relationship status?
   - ☐ Single

For peer review only - http://bmjopen.bmj.com/site/about/guidelines.xhtml

- ☐ Married
- ☐ Divorced
- ☐ Civil Partnership
- ☐ Dissolved partnership
- ☐ Co-habiting
- ☐ Separated
- ☐ In a relationship

9. Occupation/profession:

- ☐ Presently working full time
- ☐ Presently working part time
- ☐ Unemployed
- ☐ Student
- ☐ Training/apprentice

10. Indicate ALL people who live with you:

- ☐ No-one; I live alone
- ☐ Spouse/partner
- ☐ Parents
- ☐ Siblings
- ☐ Lives with others (not partner)
- ☐ Other (please specify) ____________________________

11. What is your religious preference?

- ☐ Non-religious
- ☐ Baptist
- ☐ Brethren
- ☐ Buddhist
- ☐ Catholic
- ☐ Church of Ireland
- ☐ Methodist
- ☐ Presbyterian
- ☐ Sikh
- ☐ Church of England
- ☐ Church of Scotland
- ☐ Free Presbyterian
- ☐ Hindu
- ☐ Jewish
- ☐ Muslim
- ☐ Protestant
- ☐ Other (please specify) ____________________________

For peer review only - http://bmjopen.bmj.com/site/about/guidelines.xhtml

Sexual Contact

12. Are you sexually active [for example sexual intercourse, oral sex, mutual masturbation]
   - ☐ Yes
   - ☐ No

IF YOU ANSWERED 'YES' TO QUESTION 12, PLEASE ANSWER QUESTIONS 13 – 18. IF YOU ANSWERED 'NO' PLEASE SKIP TO QUESTION 19.

13. How many male sexual partners have you had in the past 12 months:___________

14. What type of intercourse have you had in the past 12 months:
   - ☐ Anal intercourse only
   - ☐ Oral intercourse only
   - ☐ Both oral and anal intercourse

15. In the past 12 months have you used condoms (including oral sex):
   - ☐ Always
   - ☐ Sometimes
   - ☐ Never
   - ☐ Rather not say

16. Do you access sexual health services?
   - ☐Yes
   - ☐ No

   If 'No', can you give a reason as to why you don't? ___________________________

   If 'Yes', which setting do you use for sexual health?
   - ☐ GUM
   - ☐ GP
   - ☐ LGBT service provider
   - ☐ non-LGBT service provider
   - ☐ HIV Clinic
   - ☐ Other (please specify): ___________

17. Have you ever been diagnosed with a sexually transmitted infection (STI)?
   - ☐ Yes
   - ☐ No
   - ☐ Rather not say

   If yes, please provide details: ____________________________________

18. Have you ever been diagnosed/treated for genital warts?
   - ☐ Yes
   - ☐ No
   - ☐ Rather not say

19. Have you been diagnosed with HIV
   - ☐ Yes

For peer review only - http://bmjopen.bmj.com/site/about/guidelines.xhtml

☐ No
☐ Rather not say

20. Where is the first contact you usually have with people you have sex with?
☐ Bars/Clubs
☐ Saunas
☐ Websites used for sexual encounters e.g. Grindr, Gaydar, Scruff
☐ Facebook/Twitter
☐ Dating websites
☐ Through friends
☐ Other (please specify)

____

Culture

21. Is your doctor aware of your sexual orientation?
☐ Yes
☐ No
☐ Not sure

22. Would you tell a health care professional that you have sex with other men or are interested in having sex with other men in order to receive a HPV vaccination?
☐ Yes
☐ No
☐ Not sure

23. If yes, what age would you feel comfortable disclosing you had sex with men in order to receive the HPV vaccine? ____________________

24. Have you talked to your doctor or other health care professional about HPV vaccination?
☐ Yes
☐ No
☐ Rather not say

If yes, please provide details

____

25. Has a health care professional ever recommended the HPV vaccine to you?
☐ Yes
☐ No
☐ Not sure

If yes, please provide details

____

26. If you were interested in receiving the HPV vaccine, where would you feel most comfortable receiving it?

For peer review only - http://bmjopen.bmj.com/site/about/guidelines.xhtml

- ☐ GUM
- ☐ GP surgery
- ☐ LGBT service provider
- ☐ non-LGBT service provider
- ☐ HIV Clinic
- ☐ Other (please specify)

____

27. The government is considering offering the HPV vaccine to men who have sex with men at Gum/HIV Clinics/GP surgeries. What are your views on this?

____

____

____

HPV and HPV VACCINE KNOWLEDGE

Have you heard about the human papillomavirus vaccine before today?

YES ☐

NO ☐

IF YOU HAVE ANSWERED 'NO' YOU DO NOT NEED TO COMPLETE ANY FURTHER QUESTIONS IN THIS SURVEY

IF 'YES' PLEASE READ THE FOLLOWING STATEMENT AND TICK THE APPROPRIATE RESPONSE FOR YOU:

The JCVI has recommended men who have sex with men aged up to 45 years receive the HPV vaccine.

Which of the following best describes your thoughts on the HPV vaccine for **men-who-have-sex-with-men**?

I have never thought about vaccination against HPV	☐
I am undecided about vaccination against HPV	☐
I have decided and do not want to vaccinate myself against HPV	☐
I have decided to do want to vaccinate myself against HPV	☐
I have already been vaccinated against HPV	☐

Can you time how long it takes you to complete this next section of the survey?

Please answer the following questions to the best of your ability:

For peer review only - http://bmjopen.bmj.com/site/about/guidelines.xhtml

	True	False	Don't know
1. *HPV is very rare*			
2. *HPV always has visible signs or symptoms*			
3. *HPV can be transmitted through genital skin-to-skin contact*			
4. *There are many types of HPV*			
5. *HPV can cause HIV/AIDS*			
6. *HPV can cause genital warts*			
7. *Men cannot get HPV*			
8. *Using condoms reduces the chances of HPV transmission*			
9. *HPV can be cured with antibiotics*			
10. *Having many sexual partners increases the risk of getting HPV*			
11. *HPV usually doesn't need any treatment*			
12. *Most sexually active people will get HPV at some point in their lives*			
13. *Having sex at an early age increases the risk of getting HPV*			
14. *HPV can cause cancer in men*			
15. *HPV is a bacterial infection*			
16. *HPV can be transmitted through oral sex*			
17. *HPV can cause herpes*			
18. *HPV can be transmitted through anal sex*			
19. *HPV infections always lead to health problems*			
20. *A person with no symptoms cannot transmit the HPV infection*			
21. *The HPV vaccines offer protection against all sexually transmitted infections*			
22. *The HPV vaccines are most effective if given to people who've never had sex*			
23. *One of the HPV vaccines*			

For peer review only

offers protection against genital warts			
24. The HPV vaccine protects you from every type of HPV			
25. You can cure HPV by getting the HPV vaccine			

Time for completion (in minutes): ___________________________

For peer review only

Journal of Adolescent Health Kesten et al.

HPV AND HPV VACCINE ATTITUDE

Can you time how long it takes you to complete this next section of the survey?

For each statement, please indicate how much you disagree or agree by selecting the appropriate number :							
	Strongly Disagree 1	***Disagree 2***	***Somewhat Disagree 3***	***Neutral 4***	***Somewhat Agree 5***	***Agree 6***	***Strongly Agree 7***
1. I feel that the HPV vaccine will protect my sexual health.							
2. I feel that the HPV vaccine is effective in preventing HPV.							
3. I feel that the HPV vaccine is effective in preventing genital warts.							
4. I feel that the HPV vaccine is effective in preventing HPV-related cancers.							
5. I feel that vaccinating against HPV would protect my current/future partner from getting infected with HPV.							
6. I feel that it would be serious if I contracted HPV.							
7. I feel that it would be serious if I contracted genital warts.							
8. I feel that it would be serious if I contracted an HPV-related cancer.							
*9. I feel that doctors/health care providers believe vaccinating **men-who-have-sex-with-men** against HPV is a good idea.*							
10. I feel that the government believes I should vaccinate myself against HPV.							
11. The opinion of doctors/health care providers about getting the HPV vaccine matters to me.							
*12. I trust the government's opinion concerning the HPV vaccine for **men-***							

For peer review only - http://bmjopen.bmj.com/site/about/guidelines.xhtml

who-have-sex-with-men.							
13. I trust scientific evidence concerning the HPV vaccine.							
14. I have heard that the HPV vaccine is unsafe.							
15. I feel that the HPV vaccine might cause short term side-effects like pain or discomfort.							
16. I feel that the HPV vaccine is being pushed to make money for pharmaceutical companies.							
17. I feel that getting the HPV vaccine would be like performing an experiment on me.							
18. I feel that the HPV vaccine would encourage me to have sex with more partners.							
19. I feel that vaccinating for HPV would mean that I would not have to use safe sex practices.							
20. I feel that the HPV vaccine may lead to long-term health problems.							
21. I feel that the HPV vaccine may affect my fertility.							
22. I feel that getting the HPV vaccine would take too much effort.							
23. I feel that it is hard to find a clinic that would be easy to access for getting the HPV vaccine.							
24. I feel that I do not have enough information about the HPV vaccine.							
25. I feel that there has not been enough research done on the HPV vaccine.							
26. I feel that I am uncomfortable discussing my sexual health with a doctor/health care provider.							
27. I feel that I am uncomfortable talking							

For peer review only - http://bmjopen.bmj.com/site/about/guidelines.xhtml

about the HPV vaccine.							
28. I feel that the process of actually getting the HPV vaccine would be easy.							
29. I feel that the HPV vaccine requires too many doses.							
30. I feel that vaccines are a good way to protect public health.							
31. I do not like the idea of vaccines.							
32. I feel that doctors give out too many vaccines.							
33. I feel that 16 is too young to receive the HPV vaccine.							

Time for completion (in minutes): ___________________________

For peer review only - http://bmjopen.bmj.com/site/about/guidelines.xhtml

Supplementary material B. Young MSM interview/focus group topic guide

1. Introduction *(5-10 min)*

Explain purpose of focus group

Before we begin I'm going to give some background to the study, an overview of the study's aim, the purpose of this focus group and details of who is funding the work.

In this focus group/interview we are going to be discussing Human papillomavirus (HPV) which is a very common infection involved in most cervical cancers. It is transmitted via skin-to-skin contact, most commonly during sexual activity. A vaccine has been developed that protects against this infection (A.S. Forster et al. / Vaccine 30 (2012) 4505–4510). It has recently been recommended that men who have sex with men (MSM) are offered this vaccination.

There are two purposes of this study. Firstly we want to understand the knowledge and attitudes of young (16-24 years) MSM towards the HPV vaccination. Secondly we are trying to identify ways to support young MSM to have this vaccination. We are particularly interested in how to support young MSM as the HPV vaccination is expected to provide greatest protection if it is given before the first sexual encounter.

The aim of this interview/focus group is to explore your knowledge and attitudes towards this vaccine, to identify things which may encourage or discourage vaccination and possible strategies to support vaccination uptake.

These interviews will contribute towards recommendations for any efforts to support the targeted vaccination of MSM, particularly those younger than 24 years of age.

This study has been funded by Cancer Research UK.

Explain audio recording procedures

Before we get started, I'd like to tell you that I will be recording the conversation to help us remember what we discussed and so that verbatim quotes can be used in future publications. You can ask for the recording to be stopped at any time and you can stop participating at any time without having to give a reason. What you say will be kept confidential and anonymous.

Guidelines for focus groups only

- Honesty – no right or wrong answers. Everyone's experiences and opinions are important. Feel free to agree or disagree with the views of others in the group.
- Confidentiality – We want people to feel comfortable about sharing potentially sensitive information so please do not discuss what is said during the group with others outside.
- Respect – you may not agree with what is said by others in the group but it is important to show respect to each other and to allow everyone a turn to express their opinions.
- Audio recording – Where possible please try to ensure that only one person is speaking at a time to aid the audio recording and transcription.

Ensure participant(s) has(ve) copy of participant information sheet

Answer any questions

For peer review only - http://bmjopen.bmj.com/site/about/guidelines.xhtml

Complete consent form(s) and check they have been completed correctly

Commence audio recording

2. Names and ice breaker for focus groups only *(2-5 min)*
For the benefit of the audio recorder can you start by saying your name and what you are currently studying/what your occupation is?

3. Sources of information and advice *(10 mins)*
To begin I'd like us to discuss where you got or would get information / advice about sexual health issues before engaging in any form of sexual activity with another man. This includes kissing, masturbation/hand jobs, oral sex and anal sex.

Where did you / would you receive or look for sexual health information and advice?

What are your reasons for choosing these places to find information or advice?

For which types of sexual activities are you most likely to seek advice?

Did you / would you consider speaking to a healthcare professional, including GPs, university health services or GUM clinics?

What are your reasons for doing / not doing this?

Could the information or advice you received or are currently receiving have been improved at all?

4. Perceptions of HPV risk *(5-10 mins)*
Now I'd like us to talk a little bit about your sexual health concerns in terms of Sexually Transmitted Diseases.

Firstly, what Sexually Transmitted Diseases do you know of?

I have a set of cards with Sexually Transmitted Diseases on (HPV, Hep A, Hep B, Hep C, Syphilis, HIV, Gonorrhoea, Genital Warts etc.) and I'd like you to order them in terms of what you are most to least concerned about as a group.

TAKE A PHOTO OF THE ORDER.

Can you talk me through your reasons for ordering the concerns like this?

5. Attitude towards HPV vaccination *(30 mins)*

For peer review only - http://bmjopen.bmj.com/site/about/guidelines.xhtml

Journal of Adolescent Health Kesten et al.

I'd like us to move on now to discuss your views about being offered the HPV vaccination. The vaccine is most protective if it is received prior to first sexual encounter as this represents a potential exposure to HPV. Before being offered the vaccination it is likely that you would be asked to reveal your sexual orientation to a healthcare professional.

Thinking back to when you first disclosed your sexual orientation to someone, who did you disclose to?

Has any healthcare professional ever asked you about your sexual orientation?

If yes, did this happen before or after you had sex with another man?

If yes, how did the healthcare professional ask you for this information?

What were the circumstances in which the healthcare professional asked you for this information?

Could the way this information was asked have been improved at all? If yes, how?

If no, how happy would you be you to disclose your sexual orientation to a healthcare professional? What are your reasons for this?

Are there any types of healthcare professional that you would feel more comfortable disclosing your sexual orientation to than others (e.g. school nurse, GP, GUM clinic staff)?

What do you think is the best way for healthcare professionals to identify young (16-24 years) MSM who may be eligible for a HPV vaccine?

Prompts to be used if necessary:

- Through parents e.g. letters home to parents through school
- In private without parents/guardians
- Using a written questionnaire given in healthcare setting from 12/13 onwards?
- Face-to-face
- Via community LGBT organisations?

Who would you prefer to offer the HPV vaccination to you (e.g. GP, GUM clinic, school nurse etc.)?

Has anyone been offered or requested the HPV vaccine? (e.g. privately)

For what reason do you think you were offered/did you request the HPV vaccine?

How would you react to being offered the HPV vaccine?

How willing would you be to go and ask to have the HPV vaccine?

What things might prevent you or make you less likely to ask for/ accept the HPV vaccination?

What things might encourage you or make you more likely to ask for/accept the HPV vaccination?

For peer review only - http://bmjopen.bmj.com/site/about/guidelines.xhtml

Journal of Adolescent Health Kesten et al.

6. Strategies to support the introduction of HPV vaccination in MSM *(30 mins)*

In the last set of questions we'd like to discuss your views on the best approach to encouraging the uptake of HPV vaccination in young MSM.

How could we increase young MSM awareness of the need to receive the HPV vaccination?

How could young MSM be encouraged to take up the HPV vaccine?

Prompts

- Awareness campaigns through schools, GUM clinics, social media etc.

7. Close *(2-3 mins)*

That is the end of my questions. Before we finish is there anything I haven't covered today that you would like to add?

We would like you to read our interpretation of the focus group. This shall be done by us sending you an email summary of the group discussion. We would like you to let us know if you feel it is an accurate interpretation of what was discussed. If you would like to do this, please provide us with an email address. This will not be kept confidential, and only used for this purpose.

End audio recording

Thank participant(s) and answer questions.

For peer review only

For peer review only - http://bmjopen.bmj.com/site/about/guidelines.xhtml

No	Item	Guide questions/description	Response and/or page no.
**Domain 1: Research team and reflexivity**			
Personal Characteristics			
1.	Interviewer/facilitator	Which author/s conducted the interview or focus group?	CF conducted the focus groups – page 7
2.	Credentials	What were the researcher's credentials? *E.g. PhD, MD*	CF has a PhD
3.	Occupation	What was their occupation at the time of the study?	Research fellow
4.	Gender	Was the researcher male or female?	Female
5.	Experience and training	What experience or training did the researcher have?	CF had qualitative research experience through her PhD
Relationship with participants			
6.	Relationship established	Was a relationship established prior to study commencement?	CF arranged the focus groups with

For peer review only

No	Item	Guide questions/description	Response and/or page no.
			participants
7.	Participant knowledge of the interviewer	What did the participants know about the researcher? e.*g. personal goals, reasons for doing the research*	Participants were informed of the research projects objectives. The participants knew that a researcher from Queen's University Belfast was conducting the focus groups.
8.	Interviewer characteristics	What characteristics were reported about the interviewer/facilitator? e.g. *Bias, assumptions, reasons and interests in the research topic*	None
**Domain 2: study design**			
Theoretical framework			
9.	Methodological orientation and Theory	What methodological orientation was stated to underpin the study? *e.g. grounded theory, discourse analysis, ethnography, phenomenology, content analysis*	The research took an applied methodological orientation, aligning most closely with grounded theory, rather than a more

For peer review only

No	Item	Guide questions/description	Response and/or page no.
			formal approach.<br><br>Page 8
Participant selection			
10.	Sampling	How were participants selected? e.g. *purposive, convenience, consecutive, snowball*	Convenience and snowball<br><br>Page 7
11.	Method of approach	How were participants approached? e.g. *face-to-face, telephone, mail, email*	A range of approaches were used. Potential participants were provided with written information both face-to-face at community LGBTQ advocacy groups and the study was advertised online<br><br>Page 7
12.	Sample size	How many participants were in the study?	17
13.	Non-participation	How many people refused to participate or	1 person left the focus

For peer review only

No	Item	Guide questions/description	Response and/or page no.
		dropped out? Reasons?	group before it began due to time constraints.<br><br>Due to the nature of the recruitment method we do not know how many people received the participant information sheet but did not participate. No one refused to participate to the researcher.<br><br>Table 1
Setting			
14.	Setting of data collection	Where was the data collected? e.g. *home, clinic, workplace*	LGQTQ advocacy groups and a University Student's Union<br><br>Page 7
15.	Presence of non-participants	Was anyone else present besides the participants and researchers?	No

For peer review only

No	Item	Guide questions/description	Response and/or page no.
16.	Description of sample	What are the important characteristics of the sample? *e.g. demographic data, date*	Table 1
Data collection			
17.	Interview guide	Were questions, prompts, guides provided by the authors? Was it pilot tested?	The interview topic guide is described on page 7.<br><br>The topic guide was not pilot tested but the questions were applied flexibility in a semi-structured manner.<br><br>Supplementary material B.
18.	Repeat interviews	Were repeat interviews carried out? If yes, how many?	No
19.	Audio/visual recording	Did the research use audio or visual recording to collect the data?	Yes, encrypted digital audio recording equipment.<br><br>Page 8
20.	Field notes	Were field notes made during and/or after the	Yes, field notes were

For peer review only

No	Item	Guide questions/description	Response and/or page no.
		interview or focus group?	made following each focus group.
21.	Duration	What was the duration of the interviews or focus group?	The focus group length averaged 44.34 minutes (range=40.4-50.4)<br><br>Page 9
22.	Data saturation	Was data saturation discussed?	Yes<br><br>Page 7 and 14
23.	Transcripts returned	Were transcripts returned to participants for comment and/or correction?	No
**Domain 3: analysis and findings**z			
Data analysis			
24.	Number of data coders	How many data coders coded the data?	JK and CF independently coded the first transcript systematically, line-by-line, compared their coding and reached consensus

For peer review only

No	Item	Guide questions/description	Response and/or page no.
			Page 8
25.	Description of the coding tree	Did authors provide a description of the coding tree?	No
26.	Derivation of themes	Were themes identified in advance or derived from the data?	Derived from the data<br><br>Page 8
27.	Software	What software, if applicable, was used to manage the data?	QSR NVivo 10.0<br><br>Page 8
28.	Participant checking	Did participants provide feedback on the findings?	No
Reporting			
29.	Quotations presented	Were participant quotations presented to illustrate the themes / findings? Was each quotation identified? e.g. *participant number*	Yes, quotations are presented with unique identifiers
30.	Data and findings consistent	Was there consistency between the data presented and the findings?	Yes
31.	Clarity of major themes	Were major themes clearly presented in the findings?	Yes
32.	Clarity of minor	Is there a description of	Yes

For peer review only

No	Item	Guide questions/description	Response and/or page no.
	themes	diverse cases or discussion of minor themes?	

For peer review only

BMJ Open

A mixed-methods study in England and Northern Ireland to understand young men-who-have-sex-with-men's knowledge and attitude towards human papillomavirus vaccination

Journal:	*BMJ Open*
Manuscript ID	bmjopen-2018-025070.R1
Article Type:	Research
Date Submitted by the Author:	15-Mar-2019
Complete List of Authors:	Kesten, Joanna; NIHR Health Protection Research Unit in Evaluation of Interventions and NIHR CLAHRC West, University of Bristol, Bristol Medical School, Population Health Sciences<br>Flannagan, Carrie; Ulster University , Institute of Nursing and Health Research, School of Nursing<br>Ruane-McAteer , Eimear ; Queen's University Belfast, School of Nursing and Midwifery, Medical Biology Centre<br>Merriel, Samuel; University of Bristol, Centre for Academic Primary Care<br>Nadarzynski, Tom; University of Westminster, School of Social Sciences<br>Shapiro, Gilla; McGill University, Department of Psychology<br>Rosberger, Zeev; McGill University, Department of Psychology<br>Prue, G; Queen's University Belfast, School of Nursing and Midwifery, Medical Biology Centre
**Primary Subject Heading**:	Sexual health
Secondary Subject Heading:	Qualitative research
Keywords:	vaccination, HPV, sexual health, adolescent, attitudes

SCHOLARONE™
Manuscripts

A mixed-methods study in England and Northern Ireland to understand young men-who-have-sex-with-men's knowledge and attitude towards human papillomavirus vaccination

Author names

Joanna May Kesten PhD[a,b], Carrie Flannagan PhD[c], Eimear Ruane-McAteer PhD[d], Samuel W D Merriel MSc[e], Tom Nadarzynski PhD[f], Gilla K. Shapiro MPA/MPP[g], Zeev Rosberger, PhD[g], Gillian Prue PhD[d]

Author affiliations

[a] The National Institute for Health Research Health Protection Research Unit in Evaluation of Interventions, School of Social and Community Medicine, University of Bristol, UK

[b] The National Institute for Health Research Collaboration for Leadership in Applied Health Research and Care West (NIHR CLAHRC West) at University Hospitals Bristol NHS Foundation Trust, UK

[c] Institute of Nursing and Health Research, School of Nursing, Ulster University, Newtownabbey BT37 0QB c.flannagan@ulster.ac.uk

[d] School of Nursing and Midwifery, Medical Biology Centre, Queen's University Belfast, UK, BT9 7BL, g.prue@qub.ac.uk, eruanemcateer01@qub.ac.uk

[e] Centre for Academic Primary Care, University of Bristol, 39 Whatley Road Clifton Bristol BS8 2PS UK sam.merriel@bristol.ac.uk

[f] University of Westminster, School of Social Sciences. T.Nadarzynski@westminster.ac.uk

[g] Department of Psychology, McGill University, 2001 McGill College Avenue, Montreal, Quebec, Canada, H3A 1G1, gilla.shapiro@mail.mcgill.ca; zeev.rosberger@mail.mcgill.ca

For peer review only - http://bmjopen.bmj.com/site/about/guidelines.xhtml

Corresponding author details

Joanna May Kesten

Email: Jo.Kesten@bristol.ac.uk

Telephone: 0117 33 10008

Address: NIHR CLAHRC West, 9th Floor, Whitefriars, Lewins Mead, Bristol BS1 2NT

Funding statement

This study was funded by an Innovation award (#22091) from Cancer Research UK/BUPA Foundation. JK is partly funded by National Institute for Health Research (NIHR) Collaboration for Leadership in Applied Health Research and Care West (CLAHRC West) at University Hospitals Bristol NHS Foundation Trust and NIHR Health Protection Research Unit in Evaluation of Interventions. SM was the recipient of an academic clinical fellowship from NIHR. GS was supported by the Vanier Canada Graduate Scholarship and Queen Elizabeth II Diamond Jubilee Scholarship programmes.

Competing interests: None declared.

Word count: 4421

For peer review only

Abstract

Objectives: Men-who-have-sex-with-men (MSM) are at greater risk for HPV-associated cancers. Since 2016, MSM have been offered the HPV vaccination, which is most effective when received prior to sexual debut, at Genitourinary Medicine clinics in the United Kingdom. In September 2019, the national HPV vaccination programme will be extended to boys. This study aimed to understand young MSM's (YMSM) knowledge and attitudes towards human papillomavirus (HPV) vaccination.

Design: Questionnaires assessed YMSM demographics, sexual behaviour, culture, knowledge and attitudes towards HPV vaccination, and stage of vaccine decision-making using the Precaution Adoption Process Model. Focus groups explored sexual health information sources, attitudes, barriers and facilitators to vaccination, and strategies to support vaccination uptake. Questionnaire data were analysed using descriptive statistics and focus group data were analysed thematically.

Setting: Questionnaires were also completed online. Focus groups were conducted within Lesbian Gay Bisexual Trans Queer (LGBTQ) organisational settings and a university student's union in England and Northern Ireland.

Participants: Seventeen YMSM (M=20.5 years) participated in four focus groups and 51 (M=21.1 years) completed questionnaires.

Results: Over half of YMSM were aware of HPV (54.9%), yet few (21.6%) had previously discussed vaccination with a Healthcare Professional (HCP). Thematic analyses found YMSM were willing to receive the HPV vaccine. Vaccination programmes requiring YMSM to request the vaccine, particularly prior to sexual orientation disclosure to family and friends, were viewed as unfeasible. Educational campaigns explaining vaccine benefits were indicated as a way to encourage uptake.

Conclusions: This study suggests that to effectively implement HPV vaccination for YMSM, this population requires clearer information and greater discussion with their HCP. In support of the decision made by the JCVI, universal vaccination is the most feasible and equitable option. However,

For peer review only - http://bmjopen.bmj.com/site/about/guidelines.xhtml

the absence of a catch-up programme will leave a significant number of YMSM at risk of HPV infection.

Strengths and limitations of this study

- This is the first study in the UK to explore YMSM's knowledge and attitudes toward HPV vaccination.
- Use of a theoretical model of behaviour change facilitates clear conceptualisation of health behaviour change and YMSM's stage of HPV vaccine decision making.
- The qualitative component obtained a diverse range of views of YMSM in England and Northern Ireland.
- Survey findings should be interpreted with caution due to the sample size.

Keywords

HPV, sexual health, adolescent, attitudes, vaccination

For peer review only

Introduction

Human papillomavirus (HPV), the most common sexually transmitted infection (STI) worldwide [1], has serious health consequences for men and women. HPV is recognised as a causative agent in cervical cancer, and is associated with anogenital tumours, oropharyngeal cancers, and genital warts [2]. While boys and girls aged 12-13 years are vaccinated in school in Australia [3 4], the current United Kingdom (UK) strategy of vaccinating all girls aged 12-13 years does not protect young men-who-have-sex-with-men (YMSM) against HPV infection and related diseases [5] as they will not benefit from herd immunity [6].

A Joint Committee on Vaccination and Immunisation's (JCVI) statement on MSM HPV vaccination [7] in 2015 recommended that vaccination programmes be extended to MSM aged up to 45 years via Genitourinary Medicine (GUM) clinics. Mathematical modelling suggested that for MSM aged 40 or over, HPV vaccination in GUM clinics was likely to be an effective and cost-effective method of reducing HPV-related disease burden in MSM in England [8] and elsewhere. Northern Ireland, Scotland and Wales are currently offering the HPV vaccine to MSM attending GUM clinics. Following a pilot programme in England[9] which found suboptimal uptake (45%) and did not report completion rates, vaccination is now offered in GUM clinics. Hence it is important to assess the reasons why MSM might not be willing to accept the vaccine through targeted HPV vaccination. An interim statement in July 2017 suggested that given the current high uptake in females, extending immunisation to all adolescent males is "highly unlikely to be cost effective in the UK" (p13) [10 11]. In July 2018, the JCVI recommended that the national HPV vaccination programme should be extended to include adolescent boys. It is planned that the programme, beginning in September 2019, will include boys aged 12/13 (England, school year 8; Northern Ireland, school year 9). Although some may now query the importance of the MSM programme (particularly for YMSM), this will still be valid for a number of years, particularly as the government have indicated that they will not initiate a catch-up programme for boys so there are still a significant number who will remain unprotected. Indeed, it is worth noting

For peer review only - http://bmjopen.bmj.com/site/about/guidelines.xhtml

that it took 5 years of deliberation by the JCVI to make this decision and that boys aged 13 plus will not be offered the vaccine in schools.

The absence of a catch-up vaccination programme leaves many UK YMSM without funded access to the HPV vaccine before exposure to HPV[12]. There is often a delay between the age of first sexual contact with another man and disclosure of sexual orientation to a healthcare professional (HCP)[13], as a result, it is likely that MSM will have multiple sexual partners before attending a GUM clinic resulting in increased risk of HPV acquisition[14].

A systematic review found that MSM HPV vaccine knowledge was low and MSM did not consider themselves at risk of infection, although over half would accept the vaccine if offered[12]. Most of these studies were conducted in North America (and none in the UK), with MSM over 26 years of age. Minimal attention has been given to the knowledge and attitudes toward HPV vaccination among adolescent and YMSM (aged 16-24 years). This is an important area for research because MSM may acquire HPV at a young age, close to their sexual debut (the age of which is decreasing)[14]. This study aimed to examine the knowledge and attitudes of UK YMSM towards HPV vaccination to inform policy and practice recommendations for accessing this hard to reach group, supporting vaccination uptake and the optimisation of protection from HPV. Despite the changes to the vaccination programme made since this research, in the absence of a catch-up programme, the newly implemented universal programme will cover not all YMSM. Therefore, understanding YMSM knowledge and attitudes to HPV remains relevant in the UK. Our findings are also relevant for guiding other programmes internationally that do not have a gender neutral programme and are considering implementation of a programme for YMSM.

Methods

For peer review only - http://bmjopen.bmj.com/site/about/guidelines.xhtml

Study design

We conducted questionnaires and focus groups with YMSM aged 16-24 years. The two sub-studies are described separately below.

Questionnaire study

Data collection

The survey was administered online using Survey Monkey and advertised via various Lesbian Gay Bisexual Transgender Queer (LGBTQ) organisations on social media (Twitter and Facebook). We have combined both the online and pen and paper completions for this paper.

Measures

The questionnaires (Supplementary Material A) assessed demographics (adapted from Hickson *et al.*)[15]; sexual behaviour (adapted from Sadlier *et al.*)[16]; culture (adapted from Zou *et al.*) [17]; and HPV vaccine stage of decision making using the Precaution Adoption Process Model (PAPM) [18]. The PAPM has six stages of behaviour change decision-making and has been used to examine knowledge and attitudes to HPV vaccination[19]. Those who indicated awareness of the HPV vaccine, were asked to complete validated HPV knowledge/attitudes scales [20 21].

Patient and Public Involvement

The HPV knowledge/attitude questionnaire scales were adapted for use with MSM through consultation with an Expert Panel including a key stakeholder group (The Rainbow Project [TRP]) and MSM focus groups.

YMSM were not involved in the development of the qualitative component of this study, however staff from TRP helped develop the study design and documentation.

The findings will be disseminated to YMSM via social media and TRP.

For peer review only - http://bmjopen.bmj.com/site/about/guidelines.xhtml

Focus group study

Data collection

We aimed to achieve data saturation[22] by recruiting 8-10 YMSM per focus group with a mix of social background, age, ethnicity, and religion. YMSM was defined through self-identification as male (including transgender male), at or over the age of sexual consent, sexually attracted to men, or had sex with a man [14]. Age inclusion criteria were based on the World Health Organisation's definition of "young": 15 – 24 years. A minimum of 16 years was specified as it is the age of sexual consent in the UK.

For the focus groups, potential participants were provided with written study information, and asked to register their interest at local LGBTQ organisations, university information days, university student union clubs and societies, and secondary school LGBTQ groups. Organisations advertised the study through social media and snowball sampling was employed.

CF conducted the focus groups within LGBTQ organisational settings and a university student's union building.

Prior to the focus groups, participants were asked to complete the questionnaire (described above).

The focus group topic guide (Supplementary Material B) was applied flexibly to allow for emergent issues and began by exploring sources of sexual health information and advice before engaging in sexual activity (not presented here). Perceptions of HPV risk in relation to six other STI's were then discussed using a sorting task in which a list of STIs were ordered by what is least to most concerning (findings not reported here). Attitudes towards HPV vaccine, barriers and facilitators to vaccination and possible intervention strategies to support vaccination uptake were explored. Experiences of disclosing sexual orientation to HCP were also discussed. All participants were informed that the HPV vaccine was most protective if received prior to first sexual encounter. Participants were asked to reflect as to how they would have viewed taking the vaccine when they were 12-13 years.

For peer review only - http://bmjopen.bmj.com/site/about/guidelines.xhtml

For peer review only

Analysis

Questionnaire study

Questionnaire data was inputted to SPSS v12 and analysed descriptively with frequencies and proportions reported for categorical data and mean and standard deviation for continuous data. Due to lack of statistical power it was not possible to utilise inferential statistics for analysis. Participants' PAPM vaccine decision-making stage was classified into six stages: unaware, unengaged, undecided, decided not to vaccinate, decided to vaccinate and those who had already been vaccinated [19]. If participants indicated they were not sexually active they were asked to skip the sexual contact questions. If they indicated that they had never heard of the HPV vaccine they did not complete the knowledge/attitude scores. Knowledge and attitudes held by participants about HPV and HPV vaccination were analysed using descriptive statistics.

Focus group study

Focus groups were audio recorded, transcribed verbatim, anonymised and analysed thematically[23] using QSR NVivo (version 10.0). This approach was chosen because it offers a clear analysis process while remaining flexible[23]. JK and CF independently coded the first transcript systematically, line-by-line, compared their coding and reached consensus on the definition of codes. These initial codes, which captured features of interest in the data, were then applied to the remaining transcripts. The content of all the codes was read and compared to each other to iteratively refine and cluster codes into themes and sub-themes. For example, duplicate codes with synonymous meanings were collapsed. A description of each theme capturing instances of divergence was then written by JK. At each stage, findings were verified and discussed by the research team to assess accuracy and credibility of the interpretation, promote inter-rater reliability and ensure rigour.

For peer review only - http://bmjopen.bmj.com/site/about/guidelines.xhtml

Ethical approval was granted from the Queens University Belfast, School of Nursing and Midwifery Research Ethics Committee (39.GPrue.05.16.M8.V2). Written informed consent from each participant was obtained prior to participation. Participants were not provided any financial remuneration for their time.

Results

Participant characteristics

Between September 2016 and March 2018, questionnaires were completed by 51 YMSM. From this 51, four focus groups in Northern Ireland (*n*=3) and England (*n*=1) were conducted between September and December 2016 with 17 YMSM who had completed the questionnaires (Table 1). Focus group size ranged from 2-6 participants and lasted approximately 45 minutes.

Table 1. Participant characteristics

Questionnaire results

The majority (*n*=49) were sexually active and reported both oral and anal intercourse in the past 12 months (*n*=35), a wide range of partner numbers (*M*= 5 partners, range 0-25), and ‘sometimes’ (*n*=17) or ‘never’ (*n*=16) used condoms. Twenty-nine (57%) participants had accessed sexual health services (Table 2).

Nineteen participants (37%) had never heard of HPV and did not complete the rest of the questionnaire. Of those who had heard of HPV in accordance with the PAPM, 18% were in the

For peer review only - http://bmjopen.bmj.com/site/about/guidelines.xhtml

‘decided to act’ stage of vaccine decision-making (stage 5), none had decided that they did not want the vaccine (stage 4), and 22% had already been vaccinated (stage 6) (Table 3).

Of those who were aware of HPV (*n*= 28), knowledge of HPV, and the HPV vaccine was generally high; mean items correct 65% (*M*= 13.3, SD 4.7) and 60% (*M*= 3.3, SD, 1.2), respectively. However, there was wide variation in knowledge scores (HPV range, 3-20; HPV vaccine range, 0-5) (Table 3). Participants were aware that HPV affected men, the method of HPV transmission, and that vaccination was most effective if given prior to sexual debut. However, awareness of the link between HPV and genital warts and the severity of an HPV infection was lower as the majority of YMSM thought HPV infection always required treatment and that infection with HPV would always lead to health problems (Table 3).

Thirty-three participants (65%) reported that HPV vaccination had never been discussed with or recommended by a HCP (Table 3). The mean age participants were willing to disclose their sexuality to a HCP was 18.3 years (range=11-23, *SD*=2.40) (Table 3). The most comfortable setting cited to receive the HPV vaccine was primary care or LGBTQ-specific services, rather than GUM clinics (Table 3).

Table 2. Sexual contact and relationships

Table 3. HPV vaccine: culture, awareness and stage of decision making

Qualitative results

For peer review only - http://bmjopen.bmj.com/site/about/guidelines.xhtml

Two main themes and several subthemes were elicited from the thematic analysis: 1) Willingness to be vaccinated and; 2) Implementation recommendations. Anonymous quotes illustrating the key themes are presented below. Minimal differences in attitudes towards HPV between geographical settings were found.

1. Willingness to be vaccinated

Despite a perceived lack of knowledge about HPV and the vaccine and the threat posed to men, most participants were willing to receive the vaccine and wanted more information.

P1: I only knew about it because of the cervical cancer (…)

P2: I didn't even know that was what it was for.

P1: I didn't know even if like that would apply to us, so I don't even know what the dangers are.

Focus group 2

Participants were motivated to receive the vaccine to protect their health and a small number of participants suggested that the cost and number of doses of the vaccine were not barriers to vaccination.

I'm not going to say like get rid of worry because you still have to…it's your sexual health, but it would be safer in a sense (…) I'm better protected – I think would be a better way of putting it. So, I think my own health would encourage me more [to ask or accept the HPV vaccine]. I'd rather be protected than not protected.

For peer review only - http://bmjopen.bmj.com/site/about/guidelines.xhtml

Focus group 3, unidentifiable speaker

2. Implementation recommendations

Across the focus groups, recommendations to support the implementation of the HPV vaccine were gathered and grouped into two subthemes: 'Promoting and raising awareness of the vaccine' and 'Identifying and offering YMSM the HPV vaccination'.

Promoting and raising awareness of the vaccine

Better understanding of the benefits and side-effects of the vaccine were expected to encourage uptake. To promote the vaccine and inform YMSM, awareness campaigns and advertisements on the internet, radio, TV, social media, in University society's, LGBTQ youth groups and dating apps were suggested.

> *For this generation particularly, social media and TV ads and newspapers – well, probably not newspapers, but radio ads as well. You know, a campaign around getting people vaccinated, I think that would be very beneficial for young people these days.*
>
> Focus group 3, unidentifiable speaker

Participants suggested including information about the vaccine for YMSM in primary care and the sexual health education curriculum in schools. Indeed, it was noted that there is a lack of MSM-specific sexual health and relationship information provided in the latter.

> *When you're receiving that [heterosexual relationship education] in school, (...) it just reinforces the fact that you're (...) not relating to it means that you're not normal like everyone*

For peer review only - http://bmjopen.bmj.com/site/about/guidelines.xhtml

else, so you don't want to speak about it. So it would just be better if it [HPV vaccine education for MSM] was just part of that education anyway.

Focus Group 2

Identifying and offering YMSM the HPV vaccination

The ideal pre-exposure timing for vaccination and the fluid, undefined nature of sexual preferences at a young age were perceived as barriers to identifying eligible recipients. There were mixed feelings about whether it would be acceptable for HCPs to ask boys (<16 years) to disclose their sexuality for this purpose due to concern about parents being informed and a lack of a trusting relationship. It was, however, also noted that questions about sexuality need to be normalised, particularly in primary care.

Interviewer: If everybody was getting the HPV vaccine…

Participant 2: That's probably what they should do, because, I mean, (…) someone might think now, oh, I'll never have sex with a man, but then, later down the line, they might do.

Focus group 4

The focus group participants wanted the benefits of vaccination to be explained and for the vaccine to be offered in a natural, relaxed manner, opportunistically, rather than having to request it. Participants felt they would be unlikely to request the vaccine because they would need more knowledge and they felt too uncomfortable.

Participant 2: As long as there was someone professional telling me what's it about, what's it going to do, and what it could do if it goes wrong.

For peer review only - http://bmjopen.bmj.com/site/about/guidelines.xhtml

Focus group 2

Participants reckoned it was not feasible to expect young boys to identify themselves for the HPV vaccine when they potentially had not disclosed or decided their sexual orientation. There was also a preference for not singling boys out by their sexuality when offering the vaccine. Similarly, receiving the vaccine confidentially was important because the potential for bullying and embarrassment would act as barriers. It was noted by participants that universal vaccination of all boys would avoid these problems. A young person seeking sexual health advice represented an opportunity to identify eligible boys. However, this is likely to occur post-sexual encounter – after the risk of exposure to the virus.

I would want them to approach me. I wouldn't go out of my way to go and get it.

Focus group 3, unidentifiable speaker

Interviewer: So then you're asking Year 8 and 9 that age group (...) -

Unidentifiable participant: To basically out themselves…

[Agreement]

Interviewer: Do you see that as being a feasible scenario?

Unidentifiable participant: No.

Unidentifiable participant: Absolutely not.

Unidentifiable participant: The only kind of way round that is if every like male child is also vaccinated, but (...) obviously they won't do that because in terms of cost of vaccines.

Focus group 3, unidentifiable speaker

For peer review only - http://bmjopen.bmj.com/site/about/guidelines.xhtml

Participant 2: When you get your vaccinations in school, you all, (...) used to go in to get your vaccinations [as a class]. If it were like that, I wouldn't do it, because I wouldn't like anyone seeing.

Focus group 4

Participant 6: Why wouldn't it be offered to like young males in school, (...) so it was like before like presumably anybody had had sex (...). A lot more people would get it that way.

Focus group 1

There were mixed feelings about General Practitioners (GPs) or specialist sexual HCPs offering the vaccine. The relationship with the HCP was important; if YMSM have a good relationship with their GP then being offered the HPV vaccine by them is preferable. In contrast, a small number would feel more comfortable being offered the vaccine by someone they trust from a community LGBTQ group or local sexual health centre. A comment was also made about the nature of the vaccine being related to sexual health meaning it made more sense/was easier to offer it via specialist services. However, prior to disclosure or sexual activity, the participants commented that boys may not engage with or know about sexual health or LGBTQ organisations so offering the vaccine in these setting may represent a barrier.

Telling your family GP you're gay before you've told your family would be a big no I think because the GP might go back and tell your parents and then out you.

Focus group 3, unidentifiable speaker

For peer review only - http://bmjopen.bmj.com/site/about/guidelines.xhtml

If you have to go and ask about it and ask for it, who would you ask because you wouldn't be able to come here [Community LGBTQ group] because you wouldn't know here existed.

Focus group 4

Written invitations from GPs offering the vaccine to eligible patients were also suggested. However, this would require boys to identify as MSM when registering or being asked about their sexuality by a HCP. A small number of accounts suggested it would be acceptable to refer patients to receive the HPV vaccine in sexual health clinics if it was not available in a GP setting. Offering the vaccine in schools when YMSM are beginning to have their first sexual encounters was suggested. Similarly, the school nurse was a trusted individual for some and therefore may be an acceptable person to provide the vaccine.

Discussion

This is the first study to examine the views of YMSM towards the HPV vaccine in the UK. Despite being sexually active and willing to disclose sexual orientation to receive the vaccine, most participants had never been recommended the HPV vaccine, suggesting that MSM are not being offered the vaccine at the most opportune time. The data also suggested that HPV knowledge in YMSM is low, with almost half of participants being unaware of HPV or the vaccine. YMSM were willing to receive the vaccine but wanted additional information about HPV and the vaccine. Given the reluctance to disclose information about sexuality to HCP (prior to disclosure to significant others), the wide range of sexual partner numbers, and lack of consistent contraceptive use, combined with the importance of supporting vaccination prior to potential exposure, the findings highlight significant barriers to MSM accessing the vaccine. Early provision of information was recommended through awareness campaigns, advertisements and the school health education curriculum. However,

For peer review only - http://bmjopen.bmj.com/site/about/guidelines.xhtml

even with enhanced awareness, programmes that rely on YMSM to present for vaccination (particularly prior to sexual orientation disclosure) were not viewed as feasible. Furthermore, preferences for GPs or specialist HCPs offering the vaccine were dependent on the relationship with the HCP. Offering the vaccine to MSM in schools was thought to be acceptable. We accept that many of these issues will now hopefully be addressed by the extension of the current female vaccination programme to boys in September 2019, although the lack of catch-up programme for boys would indicate that there is still a need for the vaccine programme to target YMSM for at least the next six years as a significant number of YMSM will be a risk of HPV infection. In addition, these findings offer insights into barriers to vaccination for YMSM which will be useful if the uptake of a universal vaccination programme is low.

Strengths and Limitations

This is the first study in the UK exploring this topic with YMSM. By conducting this research in more than one setting we can comment on the transferability of our findings; we found minimal differences in attitudes towards HPV between settings. The use of a theoretical model of behaviour change, the PAPM, also facilitates clear conceptualisation of health behaviour change and YMSM's stage of HPV vaccine decision making.

We aimed to continue data collection until saturation, however recruitment difficulties and the study timeframe meant that the decision to cease recruitment was pragmatic. The sensitivity of the topic, the hard to reach population and the lack of monetary compensation for the participant's time are possible explanations for this. Therefore, the findings must be read with caution. Those who self-selected to participate may be more comfortable with their sexuality than those who did not agree. Indeed, recruiting through LGBTQ organisations narrowed our participant pool to those engaged with these services who had disclosed their sexual orientation. The small sample size for the quantitative data resulted in a lack of statistical power to analyse data using inferential statistics and should be

For peer review only - http://bmjopen.bmj.com/site/about/guidelines.xhtml

considered in generalising beyond the study sample. Small sample sizes in research with sexual and gender minorities is a recognised limitation[24]. The interview sample age range of 16-24 years is older than the target population for the vaccine - 12-13 years. Although the participants were asked to consider how they would view the vaccine and strategies to implement it among YMSM, it is unclear whether current YMSM share similar attitudes.

Implications for research and practice

The reluctance of YMSM to discuss their sexuality with HCPs before they have disclosed to significant others has important implications for the success of an HPV vaccination programme. Previous research shows that MSM disclosing their sexuality to significant others, visiting HCPs in the past year, and previous STI diagnosis predict disclosure to a HCP[25]. In the absence of a catch-up programme for boys, additional measures to support YMSM to access the vaccine are necessary. For instance, information may need to be provided to young men outside of healthcare settings including educational contexts during sex and relationship education or HCPs may need to take an active role in opportunistically providing information during consultations for non-sexual health related matters. To support the latter, GPs and other HCPs may require additional education and training[26, 27].

Comparison to existing literature

A lack of knowledge does not appear to deter MSM willingness to be vaccinated [28]. However, MSM in this study and in others [28], desired more information. Poor knowledge of the HPV vaccine among YMSM has also been reported previously [12 29].

For peer review only - http://bmjopen.bmj.com/site/about/guidelines.xhtml

Other qualitative work with MSM has shown support for vaccinating all adolescent boys in school in part to protect against stigma arising from vaccination policies targeting MSM[30]. This would also remove the barrier of MSM having to request the vaccination, especially prior to sexual debut[27].

Our finding that MSM are unlikely to disclose sexual orientation to a HCP prior to sexual debut, has been reported elsewhere[13], suggesting that HPV vaccine programmes delivered by HCPs would be of "limited benefit"[13]. Participants in our study recommended the vaccine be offered by HCPs rather than expecting them to request it; however it is unclear whether initial reluctance to disclose sexuality would prevent vaccination uptake. The absence of a HCP's recommendation has previously been identified as a barrier to vaccination[31]. A new NHS England standard recommending "sexual orientation monitoring" whereby patients aged 16 and over are asked to disclose their sexual orientation at every face-to-face appointment may help to identify those eligible for vaccination[32]. Although this standard would not help identify those younger than 16 years who may benefit from the vaccine.

Previous research has found that most MSM have positive attitudes towards vaccinations against STIs and would be willing to receive the HPV vaccine[29 30]. However, individual and systemic barriers such as access to sexual health clinics, disclosure of sexual orientation, concern about confidentiality or belief that HPV vaccine is not effective after sexual debut, may compromise the effectiveness of vaccination strategies[30]. Additionally, perceptions that HPV is relatively uncommon and harmless may lead to low desirability of the vaccine resulting in suboptimal coverage and therefore reduced cost-effectiveness[30].

In line with our findings, awareness raising strategies are vital to HPV vaccination programme success[29 33-35]. To raise awareness and motivate vaccine uptake, a public health campaign may be

For peer review only - http://bmjopen.bmj.com/site/about/guidelines.xhtml

necessary[28]. When developing strategies for HPV vaccination programmes, stakeholders can learn from the introduction of vaccinations such as hepatitis B and should engage with the target population and co-ordinate between stakeholders to ensure consistent messages[33]. In addition, offering the HPV vaccination to MSM alongside other vaccinations and during STI screening consultations has been recommended[29 35].

Conclusions

This study suggests that UK YMSM's are willing to receive the HPV vaccine. However, the UK's current HPV vaccine programme that relies on MSM to present for vaccination (particularly prior to sexual orientation disclosure) was not viewed as feasible. The importance of supporting vaccination prior to potential virus exposure combined with the reluctance to disclose information about sexual orientation means personal knowledge and awareness of the HPV vaccine is important therefore, early provision of information is recommended. Offering the vaccine in healthcare and education settings may be acceptable, although the barriers to this channel of provision may mean that, in support of the decision made by the JCVI, universal vaccination is the most feasible and equitable option. However, in the absence of a catch-up programme there is still a need for the UK vaccine programme to target YMSM as a significant number will remain at risk of HPV infection. These findings also help guide other programmes internationally that do not have a gender-neutral programme and are considering implementation of a programme for YMSM.

For peer review only - http://bmjopen.bmj.com/site/about/guidelines.xhtml

Table 1. Participant characteristics

Participant characteristics	Questionnaire participants			Focus group participants (subset of questionnaire participants)		
	M (*SD*)	Range	*N*	*M* (*SD*)	Range	N (% of sample)
Age (years)	21.06 (2.6)	16-24		20.5 (2.73)	16-24	18[1] (100)
Ethnicity						
- White			44 (86.3)			15 (83.3)
- Other			6 (11.8)			3 (16.6)
- Missing			1 (1.9)			-
Location						
- Northern Ireland			36			13 (72.2)
- England			15			5 (27.8)
Education						
- Full-time education			26			11
- Employed full-time			17			4
- Employed part-time			5			1
- Unemployed			2			1
- Missing			1			1

For peer review only - http://bmjopen.bmj.com/site/about/guidelines.xhtml

Group size	
Focus group 1[1]	6
Focus group 2	2
Focus group 3	4
Focus group 4	5

[1]One participant completed the questionnaire and left before the focus group began due to time constraints

For peer review only

For peer review only - http://bmjopen.bmj.com/site/about/guidelines.xhtml

Table 2. Sexual contact and relationships

**Sexual contact and relationships**	***M (SD)***	**Range**	***N (%)***
Have you ever in the past had sex with a man or do you plan to in the future?			
- Yes			49 (96.08%)
- No			1 (1.96%)
- Missing			1 (1.96%)
Relationship status:			
- Single			26 (50.98%)
- In a relationship			21 (41.18%)
- Co-habiting			2 (3.92%)
- Civil Partnership			1 (1.96%)
- Missing			1 (1.96%)
Are you sexually active?			
- Yes			38 (74.51%)
- No			8 (15.69%)
- Missing			5 (9.8%)
How many male sexual partners have you had in the past 12 months?	5 (6)	0-25	
What type of intercourse have you had in the past 12 months?			
- Oral only			3 (5.88%)
- Anal only			2 (3.92%)
- Both oral and anal			35 (68.63%)
- Neither			3 (5.88%)

For peer review only

- Missing	8 (15.69%)
In the past 12 months have you used condoms?	
- Always	9 (17.65%)
- Sometimes	17 (33.33%)
- Never	16 (31.37%)
- Prefer not to say	1 (1.96%)
- Missing	8 (15.69%)
Do you access sexual health services?	
- Yes	29 (56.86%)
- No	14 (27.45%)
- Missing	8 (15.69%)

For peer review only

For peer review only - http://bmjopen.bmj.com/site/about/guidelines.xhtml

BMJ STI Kesten et al. HPV vaccination among young MSM

Table 3. HPV vaccine: culture, awareness and stage of decision making

	M (*SD*)	Range	*N*
GP aware of sexuality			
- Yes			22 (43.14%)
- No			17 (33.33%)
- Not sure			8 (15.69%)
- Missing			4 (7.84%)
Willing to disclose MSM status to HCP to receive HPV vaccine?			
- Yes			41 (80.39%)
- No			3 (5.88%)
- Not sure			3 (5.88%)
- Missing			4 (7.84%)
If yes, at what age?	18.3 (2.4)	11-23	
Has a HCP ever recommended an HPV vaccine to you?			
- Yes			11 (21.57%)
- No			33 (64.71%)
- Not sure			1 (1.96%)
- Missing			6 (11.76%)
Discussed HPV vaccination with HCP			
- Yes			10 (19.61%)
- No			34 (66.67%)
- Missing			7 (13.73%)

For peer review only - http://bmjopen.bmj.com/site/about/guidelines.xhtml

Most comfortable setting to receive HPV vaccine*some ticked more than one option*:	17 (33.33%)
- Genitourinary medicine (GUM)	30 (58.82%)
- Primary care	33 (64.71%)
- Lesbian Gay Bisexual	1 (1.96%)
Transgender organisations	2 (3.92%)
- Non-LGBTQ specific sexual	1 (1.96%)
health provider	
- HIV clinic	
Prior awareness of HPV	
- Yes	28 (54.9%)
- No	19 (37.25%)
- Missing	4 (7.84%)
PAPM (Stage of vaccine decision making)	
- *Stage 2 Unengaged*: I have never thought about vaccination against HPV	17 (33.33%)
- *Stage 3 Undecided*: I am undecided about vaccination against HPV	2 (3.92%)
- *Stage 4 Decided not to act*: I have decided and do not want to vaccinate myself against HPV	0
	9 (17.65%)

For peer review only - http://bmjopen.bmj.com/site/about/guidelines.xhtml

For peer review only

- *Stage 5 Decided to act*: I have decided and I do want to vaccinate myself against HPV			11 (21.57%)
- *Stage 6 Acted*: I have already been vaccinated against HPV			12 (23.53%)
- Missing			
Knowledge scores			
- HPV knowledge score (max 20)	13.3 (4.7)	3-20	27
- HPV vaccination knowledge score (max 5)	3.3 (1.2)	0-5	27

For peer review only

For peer review only - http://bmjopen.bmj.com/site/about/guidelines.xhtml

Contributors: JK, GP and ERM drafted the manuscript and JK led the analysis of the qualitative data supported by CF. CF conducted the focus groups and questionnaires. GP, JK and SM conceived the research question. ZR's research team developed the original questionnaires and use of the PAPM in college males and parents of young children eligible for the HPV vaccine (prior to adaption for MSM). GP led the research team. ERM and GP conducted the analysis of the questionnaire data and led the writing of these sections of the paper. All co-authors (JK, CF, ERM, SM, TN, GS, ZR, GP) developed the research question, methodology and supported the management of the project. All authors (JK, CF, ERM, SM, TN, GS, ZR, GP) have read, contributed to and approved the final manuscript.

Acknowledgement:

We would like to thank the MSM and TRP stakeholders who informed the development of the HPV knowledge/attitude questionnaire scales.

Participant consent: written consent obtained.

Ethics approval: This study was approved by the Queens University Belfast, School of Nursing and Midwifery Research Ethics Committee (39.GPrue.05.16.M8.V2).

Data sharing statement: No data are available.

For peer review only

For peer review only - http://bmjopen.bmj.com/site/about/guidelines.xhtml

BMJ STI Kesten et al. HPV vaccination among young MSM

References

1. Forman D, de Martel C, Lacey CJ, et al. Global burden of human papillomavirus and related diseases. *Vaccine* 2012;30 Suppl 5:F12-23. doi: 10.1016/j.vaccine.2012.07.055 [published Online First: 2012/12/05]
2. Liddon N, Hood J, Wynn BA, et al. Acceptability of human papillomavirus vaccine for males: a review of the literature. *The Journal of adolescent health : official publication of the Society for Adolescent Medicine* 2010;46(2):113-23. doi: 10.1016/j.jadohealth.2009.11.199 [published Online First: 2010/02/02]
3. Prevention.; CfDCa. Human Papillomavirus (HPV). Genital HPV Infection - Fact Sheet 2017 [accessed 31st October 2017.
4. Health AGDo. Immunise Australia Program Human Papillomavirus (HPV) Australian Goverment Department of Health2017 [Available from: http://www.immunise.health.gov.au/internet/immunise/publishing.nsf/Content/immunise-hpv accessed 30th November 2017.
5. Read TR, Hocking JS, Chen MY, et al. The near disappearance of genital warts in young women 4 years after commencing a national human papillomavirus (HPV) vaccination programme. *Sexually transmitted infections* 2011;87(7):544-7. doi: 10.1136/sextrans-2011-050234 [published Online First: 2011/10/06]
6. Machalek DA, Poynten M, Jin F, et al. Anal human papillomavirus infection and associated neoplastic lesions in men who have sex with men: a systematic review and meta-analysis. *The Lancet Oncology* 2012;13(5):487-500. doi: 10.1016/s1470-2045(12)70080-3 [published Online First: 2012/03/27]
7. Health.; Do, England. PH. JCVI statement on HPV vaccination of men who have sex with men 2015 [Available from: https://www.gov.uk/government/publications/jcvi-statement-on-hpv-vaccination-of-men-who-have-sex-with-men accessed 7th February 2016.
8. Lin A, Ong KJ, Hobbelen P, et al. Impact and Cost-effectiveness of Selective Human Papillomavirus Vaccination of Men Who Have Sex With Men. *Clinical infectious diseases : an official publication of the Infectious Diseases Society of America* 2017;64(5):580-88. doi: 10.1093/cid/ciw845 [published Online First: 2016/12/25]
9. Edelstein M, Iyanger N, Hennessy N, et al. Implementation and evaluation of the human papillomavirus (HPV) vaccination pilot for men who have sex with men (MSM), England, April 2016 to March 2017. 2019;24(8):1800055. doi: doi:https://doi.org/10.2807/1560-7917.ES.2019.24.8.1800055
10. Health.; Do, England. PH. JCVI statement: extending the HPV vaccination programme 2017 [Available from: https://www.gov.uk/government/publications/jcvi-statement-extending-the-hpv-vaccination-programme accessed 8th August 2017.
11. Immunisation JCoVa. Joint Committee on Vaccination and Immunisation [minute 2017 10.Draft.pdf]: Gov.UK; 2017 [Available from: https://www.gov.uk/government/groups/joint-committee-on-vaccination-and-immunisation#minutes.
12. Nadarzynski T, Smith H, Richardson D, et al. Human papillomavirus and vaccine-related perceptions among men who have sex with men: a systematic review. *Sexually transmitted infections* 2014;90(7):515-23. doi: 10.1136/sextrans-2013-051357 [published Online First: 2014/05/03]
13. Rank C, Gilbert M, Ogilvie G, et al. Acceptability of human papillomavirus vaccination and sexual experience prior to disclosure to health care providers among men who have sex with men in Vancouver, Canada: implications for targeted vaccination programs. *Vaccine* 2012;30(39):5755-60. doi: 10.1016/j.vaccine.2012.07.001 [published Online First: 2012/07/17]

For peer review only - http://bmjopen.bmj.com/site/about/guidelines.xhtml

14. Zou H, Tabrizi SN, Grulich AE, et al. Early acquisition of anogenital human papillomavirus among teenage men who have sex with men. *The Journal of infectious diseases* 2014;209(5):642-51. doi: 10.1093/infdis/jit626 [published Online First: 2013/11/23]
15. Hickson F, Tomlin K, Hargreaves J, et al. Internet-based cohort study of HIV testing over 1 year among men who have sex with men living in England and exposed to a social marketing intervention promoting testing. *Sexually transmitted infections* 2015;91(1):24-30. doi: 10.1136/sextrans-2014-051598 [published Online First: 2014/10/02]
16. Sadlier C, Rowley D, Morley D, et al. Prevalence of human papillomavirus in men who have sex with men in the era of an effective vaccine; a call to act. *HIV medicine* 2014;15(8):499-504. doi: 10.1111/hiv.12150 [published Online First: 2014/03/25]
17. Zou H, Grulich AE, Cornall AM, et al. How very young men who have sex with men view vaccination against human papillomavirus. *Vaccine* 2014;32(31):3936-41. doi: 10.1016/j.vaccine.2014.05.043 [published Online First: 2014/05/24]
18. Weinstein ND. The precaution adoption process. *Health psychology : official journal of the Division of Health Psychology, American Psychological Association* 1988;7(4):355-86. [published Online First: 1988/01/01]
19. Perez S, Tatar O, Shapiro GK, et al. Psychosocial determinants of parental human papillomavirus (HPV) vaccine decision-making for sons: Methodological challenges and initial results of a pan-Canadian longitudinal study. *BMC public health* 2016;16(1):1223. doi: 10.1186/s12889-016-3828-9 [published Online First: 2016/12/07]
20. Perez S, Shapiro GK, Tatar O, et al. Development and Validation of the Human Papillomavirus Attitudes and Beliefs Scale in a National Canadian Sample. *Sexually transmitted diseases* 2016;43(10):626-32. doi: 10.1097/olq.0000000000000506 [published Online First: 2016/09/16]
21. Perez S, Tatar O, Ostini R, et al. Extending and validating a human papillomavirus (HPV) knowledge measure in a national sample of Canadian parents of boys. *Preventive medicine* 2016;91:43-49. doi: 10.1016/j.ypmed.2016.07.017 [published Online First: 2016/07/30]
22. Glaser B, Strauss A. The discovery of grounded theory: Strategies for qualitative research. Chicago: Aldine 1967.
23. V.; B, V. C. Using thematic analysis in psychology. *Qual Res Psychol* 2006;3:77–101.
24. Fraser G. Evaluating inclusive gender identity measures for use in quantitative psychological research. *Psychology & Sexuality* 2018;9(4):343-57. doi: 10.1080/19419899.2018.1497693
25. Stupiansky NW, Liau A, Rosenberger J, et al. Young Men's Disclosure of Same Sex Behaviors to Healthcare Providers and the Impact on Health: Results from a US National Sample of Young Men Who Have Sex with Men. *AIDS patient care and STDs* 2017;31(8):342-47. doi: 10.1089/apc.2017.0011 [published Online First: 2017/07/29]
26. Merriel S, Flannagan C, Kesten J, et al. Knowledge and Attitudes of General Practitioners and Sexual Health Care Professionals Regarding Human Papillomavirus Vaccination for Young Men Who Have Sex with Men. *International Journal of Environmental Research and Public Health* 2018;15(1):151.
27. Scully D, Irving N, Daly M, et al. HPV vaccination among men who have sex with men in Ireland: GPs' awareness and vaccination rates. *Sexually transmitted infections* 2017 doi: 10.1136/sextrans-2017-053372
28. Gutierrez B, Leung A, Jones KT, et al. Acceptability of the Human Papillomavirus Vaccine Among Urban Adolescent Males. *American Journal of Men's Health* 2012;7(1):27-36. doi: 10.1177/1557988312456697
29. Fontenot HB, Fantasia HC, Vetters R, et al. Increasing HPV vaccination and eliminating barriers: Recommendations from young men who have sex with men. *Vaccine* 2016;34(50):6209-16. doi: 10.1016/j.vaccine.2016.10.075

For peer review only

For peer review only - http://bmjopen.bmj.com/site/about/guidelines.xhtml

30. Nadarzynski T, Smith H, Richardson D, et al. Perceptions of HPV and attitudes towards HPV vaccination amongst men who have sex with men: A qualitative analysis. *British Journal of Health Psychology* 2017;22(2):345-61. doi: 10.1111/bjhp.12233
31. Donahue KL, Stupiansky NW, Alexander AB, et al. Acceptability of the human papillomavirus vaccine and reasons for non-vaccination among parents of adolescent sons. *Vaccine* 2014;32(31):3883-5. doi: 10.1016/j.vaccine.2014.05.035 [published Online First: 2014/05/23]
32. Unit NEEaHI. Implementation Guidance Fundamental Standard for Sexual Orientation Monitoring 2017 [2017.
33. Sherris J, Friedman A, Wittet S, et al. Chapter 25: Education, training, and communication for HPV vaccines. *Vaccine* 2006;24 Suppl 3:S3/210-8. doi: 10.1016/j.vaccine.2006.05.124 [published Online First: 2006/09/05]
34. Brandt HM, McCree DH, Lindley LL, et al. An evaluation of printed HPV educational materials. *Cancer control : journal of the Moffitt Cancer Center* 2005;12 Suppl 2:103-6. [published Online First: 2005/12/06]
35. Apaydin KZ, Fontenot HB, Borba CPC, et al. Three-dose HPV vaccine completion among sexual and gender minority young adults at a Boston community health center. *Vaccine* 2018;36(32, Part B):4897-903. doi: https://doi.org/10.1016/j.vaccine.2018.06.057

For peer review only - http://bmjopen.bmj.com/site/about/guidelines.xhtml

Supplementary material A. Young HIMMS men-who-have-sex-with-men questionnaire

Demographic Information

1. What is your post code: ___________

2. What age are you: ___________

3. Are you:
   - ☐ Male (including trans)
   - ☐ Female (including trans)
   - ☐ Other (Non-Binary, Gender Neutral etc. Please specify): ____________

4. Do you identify as the following:
   - ☐ Gay Man
   - ☐ Bisexual
   - ☐ Man who has sex with men
   - ☐ Heterosexual
   - ☐ Unsure/Other (Please specify): ____________

5. Have you ever in the past had sex with another man or do you plan to in the future?
   - ☐ Yes
   - ☐ No
   - ☐ Unsure

   IF YOU ANSWER 'NO' TO THE ABOVE QUESTION YOU DO NOT NEED TO COMPLETE ANY FURTHER QUESTIONS.

6. Present cigarette smoking status
   - ☐ Non Smoker
   - ☐ Smoker
   - ☐ Former

7. To which ethnic group do you consider yourself to belong to?
   - ☐ Bangladeshi
   - ☐ Black African
   - ☐ Black Caribbean
   - ☐ Central European
   - ☐ Eastern European
   - ☐ Indian
   - ☐ Pakistani
   - ☐ Chinese
   - ☐ White
   - ☐ Mixed ethnicity
   - ☐ Other (please specify) _________________

8. What is your current relationship status?
   - ☐ Single

For peer review only - http://bmjopen.bmj.com/site/about/guidelines.xhtml

- ☐ Married
- ☐ Divorced
- ☐ Civil Partnership
- ☐ Dissolved partnership
- ☐ Co-habiting
- ☐ Separated
- ☐ In a relationship

9. Occupation/profession:

- ☐ Presently working full time
- ☐ Presently working part time
- ☐ Unemployed
- ☐ Student
- ☐ Training/apprentice

10. Indicate ALL people who live with you:

- ☐ No-one; I live alone
- ☐ Spouse/partner
- ☐ Parents
- ☐ Siblings
- ☐ Lives with others (not partner)
- ☐ Other (please specify) ___________________________

11. What is your religious preference?

- ☐ Non-religious
- ☐ Baptist
- ☐ Brethren
- ☐ Buddhist
- ☐ Catholic
- ☐ Church of Ireland
- ☐ Methodist
- ☐ Presbyterian
- ☐ Sikh
- ☐ Other (please specify) ____________________________
- ☐ Church of England
- ☐ Church of Scotland
- ☐ Free Presbyterian
- ☐ Hindu
- ☐ Jewish
- ☐ Muslim
- ☐ Protestant

For peer review only

For peer review only - http://bmjopen.bmj.com/site/about/guidelines.xhtml

Sexual Contact

12. Are you sexually active [for example sexual intercourse, oral sex, mutual masturbation]
    - ☐ Yes
    - ☐ No

IF YOU ANSWERED 'YES' TO QUESTION 12, PLEASE ANSWER QUESTIONS 13 – 18. IF YOU ANSWERED 'NO' PLEASE SKIP TO QUESTION 19.

13. How many male sexual partners have you had in the past 12 months:___________

14. What type of intercourse have you had in the past 12 months:
    - ☐ Anal intercourse only
    - ☐ Oral intercourse only
    - ☐ Both oral and anal intercourse

15. In the past 12 months have you used condoms (including oral sex):
    - ☐ Always
    - ☐ Sometimes
    - ☐ Never
    - ☐ Rather not say

16. Do you access sexual health services?
    - ☐Yes
    - ☐ No

    If 'No', can you give a reason as to why you don't? _________________________

    If 'Yes', which setting do you use for sexual health?
    - ☐ GUM
    - ☐ GP
    - ☐ LGBT service provider
    - ☐ non-LGBT service provider
    - ☐ HIV Clinic
    - ☐ Other (please specify): ___________

17. Have you ever been diagnosed with a sexually transmitted infection (STI)?
    - ☐ Yes
    - ☐ No
    - ☐ Rather not say

    If yes, please provide details: ___________________________________

18. Have you ever been diagnosed/treated for genital warts?
    - ☐ Yes
    - ☐ No
    - ☐ Rather not say

19. Have you been diagnosed with HIV
    - ☐ Yes

For peer review only - http://bmjopen.bmj.com/site/about/guidelines.xhtml

- ☐ No
- ☐ Rather not say

20. Where is the first contact you usually have with people you have sex with?
    - ☐ Bars/Clubs
    - ☐ Saunas
    - ☐ Websites used for sexual encounters e.g. Grindr, Gaydar, Scruff
    - ☐ Facebook/Twitter
    - ☐ Dating websites
    - ☐ Through friends
    - ☐ Other (please specify)

    ____

Culture

21. Is your doctor aware of your sexual orientation?
    - ☐ Yes
    - ☐ No
    - ☐ Not sure

22. Would you tell a health care professional that you have sex with other men or are interested in having sex with other men in order to receive a HPV vaccination?
    - ☐ Yes
    - ☐ No
    - ☐ Not sure

23. If yes, what age would you feel comfortable disclosing you had sex with men in order to receive the HPV vaccine? ______________________

24. Have you talked to your doctor or other health care professional about HPV vaccination?
    - ☐ Yes
    - ☐ No
    - ☐ Rather not say

    If yes, please provide details

    ____

25. Has a health care professional ever recommended the HPV vaccine to you?
    - ☐ Yes
    - ☐ No
    - ☐ Not sure

    If yes, please provide details

    ____

26. If you were interested in receiving the HPV vaccine, where would you feel most comfortable receiving it?

For peer review only - http://bmjopen.bmj.com/site/about/guidelines.xhtml

For peer review only

Journal of Adolescent Health Kesten et al.

☐ GUM
☐ GP surgery
☐ LGBT service provider
☐ non-LGBT service provider
☐ HIV Clinic
☐ Other (please specify)

____

27. The government is considering offering the HPV vaccine to men who have sex with men at Gum/HIV Clinics/GP surgeries. What are your views on this?

____

____

____

HPV and HPV VACCINE KNOWLEDGE

Have you heard about the human papillomavirus vaccine before today?

YES ☐

NO ☐

IF YOU HAVE ANSWERED 'NO' YOU DO NOT NEED TO COMPLETE ANY FURTHER QUESTIONS IN THIS SURVEY

IF 'YES' PLEASE READ THE FOLLOWING STATEMENT AND TICK THE APPROPRIATE RESPONSE FOR YOU:

The JCVI has recommended men who have sex with men aged up to 45 years receive the HPV vaccine.

Which of the following best describes your thoughts on the HPV vaccine for **men-who-have-sex-with-men**?

I have never thought about vaccination against HPV ☐
I am undecided about vaccination against HPV ☐
I have decided and do not want to vaccinate myself against HPV ☐
I have decided to do want to vaccinate myself against HPV ☐
I have already been vaccinated against HPV ☐

Can you time how long it takes you to complete this next section of the survey?

Please answer the following questions to the best of your ability:

For peer review only - http://bmjopen.bmj.com/site/about/guidelines.xhtml

	True	False	Don't know
1. *HPV is very rare*			
2. *HPV always has visible signs or symptoms*			
3. *HPV can be transmitted through genital skin-to-skin contact*			
4. *There are many types of HPV*			
5. *HPV can cause HIV/AIDS*			
6. *HPV can cause genital warts*			
7. *Men cannot get HPV*			
8. *Using condoms reduces the chances of HPV transmission*			
9. *HPV can be cured with antibiotics*			
10. *Having many sexual partners increases the risk of getting HPV*			
11. *HPV usually doesn't need any treatment*			
12. *Most sexually active people will get HPV at some point in their lives*			
13. *Having sex at an early age increases the risk of getting HPV*			
14. *HPV can cause cancer in men*			
15. *HPV is a bacterial infection*			
16. *HPV can be transmitted through oral sex*			
17. *HPV can cause herpes*			
18. *HPV can be transmitted through anal sex*			
19. *HPV infections always lead to health problems*			
20. *A person with no symptoms cannot transmit the HPV infection*			
21. *The HPV vaccines offer protection against all sexually transmitted infections*			
22. *The HPV vaccines are most effective if given to people who've never had sex*			

For peer review only

Journal of Adolescent Health Kesten et al.

23. One of the HPV vaccines offers protection against genital warts			
24. The HPV vaccine protects you from every type of HPV			
25. You can cure HPV by getting the HPV vaccine			

Time for completion (in minutes): ___________________________

For peer review only

HPV AND HPV VACCINE ATTITUDE

Can you time how long it takes you to complete this next section of the survey?

For each statement, please indicate how much you disagree or agree by selecting the appropriate number :							
	Strongly Disagree 1	***Disagree 2***	***Somewhat Disagree 3***	***Neutral 4***	***Somewhat Agree 5***	***Agree 6***	***Strongly Agree 7***
1. *I feel that the HPV vaccine will protect my sexual health.*							
2. *I feel that the HPV vaccine is effective in preventing HPV.*							
3. *I feel that the HPV vaccine is effective in preventing genital warts.*							
4. *I feel that the HPV vaccine is effective in preventing HPV-related cancers.*							
5. *I feel that vaccinating against HPV would protect my current/future partner from getting infected with HPV.*							
6. *I feel that it would be serious if I contracted HPV.*							
7. *I feel that it would be serious if I contracted genital warts.*							
8. *I feel that it would be serious if I contracted an HPV-related cancer.*							
9. *I feel that doctors/health care providers believe vaccinating* ***men-who-have-sex-with-men*** *against HPV is a good idea.*							
10. *I feel that the government believes I should vaccinate myself against HPV.*							
11. *The opinion of doctors/health care providers about getting the HPV vaccine matters to me.*							
12. *I trust the government's opinion concerning the HPV vaccine for* ***men-who-have-sex-with-men.***							
13. *I trust scientific evidence*							

For peer review only

For peer review only - http://bmjopen.bmj.com/site/about/guidelines.xhtml

concerning the HPV vaccine.							
14. *I have heard that the HPV vaccine is unsafe.*							
15. *I feel that the HPV vaccine might cause short term side-effects like pain or discomfort.*							
16. *I feel that the HPV vaccine is being pushed to make money for pharmaceutical companies.*							
17. *I feel that getting the HPV vaccine would be like performing an experiment on me.*							
18. *I feel that the HPV vaccine would encourage me to have sex with more partners.*							
19. *I feel that vaccinating for HPV would mean that I would not have to use safe sex practices.*							
20. *I feel that the HPV vaccine may lead to long-term health problems.*							
21. *I feel that the HPV vaccine may affect my fertility.*							
22. *I feel that getting the HPV vaccine would take too much effort.*							
23. *I feel that it is hard to find a clinic that would be easy to access for getting the HPV vaccine.*							
24. *I feel that I do not have enough information about the HPV vaccine.*							
25. *I feel that there has not been enough research done on the HPV vaccine.*							
26. *I feel that I am uncomfortable discussing my sexual health with a doctor/health care provider.*							
27. *I feel that I am uncomfortable talking about the HPV vaccine.*							
28. *I feel that the process of actually getting the HPV vaccine would be easy.*							

For peer review only - http://bmjopen.bmj.com/site/about/guidelines.xhtml

29. *I feel that the HPV vaccine requires too many doses.*							
30. *I feel that vaccines are a good way to protect public health.*							
31. *I do not like the idea of vaccines.*							
32. *I feel that doctors give out too many vaccines.*							
33. *I feel that 16 is too young to receive the HPV vaccine.*							

Time for completion (in minutes): ___________________________

For peer review only

For peer review only - http://bmjopen.bmj.com/site/about/guidelines.xhtml

Journal of Adolescent Health Kesten et al.

Supplementary material B. Young MSM interview/focus group topic guide

1. Introduction *(5-10 min)*

Explain purpose of focus group

Before we begin I'm going to give some background to the study, an overview of the study's aim, the purpose of this focus group and details of who is funding the work.

In this focus group/interview we are going to be discussing Human papillomavirus (HPV) which is a very common infection involved in most cervical cancers. It is transmitted via skin-to-skin contact, most commonly during sexual activity. A vaccine has been developed that protects against this infection (A.S. Forster et al. / Vaccine 30 (2012) 4505–4510). It has recently been recommended that men who have sex with men (MSM) are offered this vaccination.

There are two purposes of this study. Firstly we want to understand the knowledge and attitudes of young (16-24 years) MSM towards the HPV vaccination. Secondly we are trying to identify ways to support young MSM to have this vaccination. We are particularly interested in how to support young MSM as the HPV vaccination is expected to provide greatest protection if it is given before the first sexual encounter.

The aim of this interview/focus group is to explore your knowledge and attitudes towards this vaccine, to identify things which may encourage or discourage vaccination and possible strategies to support vaccination uptake.

These interviews will contribute towards recommendations for any efforts to support the targeted vaccination of MSM, particularly those younger than 24 years of age.

This study has been funded by Cancer Research UK.

Explain audio recording procedures

Before we get started, I'd like to tell you that I will be recording the conversation to help us remember what we discussed and so that verbatim quotes can be used in future publications. You can ask for the recording to be stopped at any time and you can stop participating at any time without having to give a reason. What you say will be kept confidential and anonymous.

Guidelines for focus groups only

- Honesty – no right or wrong answers. Everyone's experiences and opinions are important. Feel free to agree or disagree with the views of others in the group.
- Confidentiality – We want people to feel comfortable about sharing potentially sensitive information so please do not discuss what is said during the group with others outside.
- Respect – you may not agree with what is said by others in the group but it is important to show respect to each other and to allow everyone a turn to express their opinions.
- Audio recording – Where possible please try to ensure that only one person is speaking at a time to aid the audio recording and transcription.

Ensure participant(s) has(ve) copy of participant information sheet

Answer any questions

For peer review only - http://bmjopen.bmj.com/site/about/guidelines.xhtml

Complete consent form(s) and check they have been completed correctly

Commence audio recording

2. Names and ice breaker for focus groups only *(2-5 min)*

For the benefit of the audio recorder can you start by saying your name and what you are currently studying/what your occupation is?

3. Sources of information and advice *(10 mins)*

To begin I'd like us to discuss where you got or would get information / advice about sexual health issues before engaging in any form of sexual activity with another man. This includes kissing, masturbation/hand jobs, oral sex and anal sex.

Where did you / would you receive or look for sexual health information and advice?

What are your reasons for choosing these places to find information or advice?

For which types of sexual activities are you most likely to seek advice?

Did you / would you consider speaking to a healthcare professional, including GPs, university health services or GUM clinics?

What are your reasons for doing / not doing this?

Could the information or advice you received or are currently receiving have been improved at all?

4. Perceptions of HPV risk *(5-10 mins)*

Now I'd like us to talk a little bit about your sexual health concerns in terms of Sexually Transmitted Diseases.

Firstly, what Sexually Transmitted Diseases do you know of?

I have a set of cards with Sexually Transmitted Diseases on (HPV, Hep A, Hep B, Hep C, Syphilis, HIV, Gonorrhoea, Genital Warts etc.) and I'd like you to order them in terms of what you are most to least concerned about as a group.

TAKE A PHOTO OF THE ORDER.

Can you talk me through your reasons for ordering the concerns like this?

5. Attitude towards HPV vaccination *(30 mins)*

For peer review only - http://bmjopen.bmj.com/site/about/guidelines.xhtml

I'd like us to move on now to discuss your views about being offered the HPV vaccination. The vaccine is most protective if it is received prior to first sexual encounter as this represents a potential exposure to HPV. Before being offered the vaccination it is likely that you would be asked to reveal your sexual orientation to a healthcare professional.

Thinking back to when you first disclosed your sexual orientation to someone, who did you disclose to?

Has any healthcare professional ever asked you about your sexual orientation?

If yes, did this happen before or after you had sex with another man?

If yes, how did the healthcare professional ask you for this information?

What were the circumstances in which the healthcare professional asked you for this information?

Could the way this information was asked have been improved at all? If yes, how?

If no, how happy would you be you to disclose your sexual orientation to a healthcare professional? What are your reasons for this?

Are there any types of healthcare professional that you would feel more comfortable disclosing your sexual orientation to than others (e.g. school nurse, GP, GUM clinic staff)?

What do you think is the best way for healthcare professionals to identify young (16-24 years) MSM who may be eligible for a HPV vaccine?

Prompts to be used if necessary:

- Through parents e.g. letters home to parents through school
- In private without parents/guardians
- Using a written questionnaire given in healthcare setting from 12/13 onwards?
- Face-to-face
- Via community LGBT organisations?

Who would you prefer to offer the HPV vaccination to you (e.g. GP, GUM clinic, school nurse etc.)?

Has anyone been offered or requested the HPV vaccine? (e.g. privately)

For what reason do you think you were offered/did you request the HPV vaccine?

How would you react to being offered the HPV vaccine?

How willing would you be to go and ask to have the HPV vaccine?

What things might prevent you or make you less likely to ask for/ accept the HPV vaccination?

What things might encourage you or make you more likely to ask for/accept the HPV vaccination?

For peer review only - http://bmjopen.bmj.com/site/about/guidelines.xhtml

Journal of Adolescent Health Kesten et al.

6. Strategies to support the introduction of HPV vaccination in MSM *(30 mins)*

In the last set of questions we'd like to discuss your views on the best approach to encouraging the uptake of HPV vaccination in young MSM.

How could we increase young MSM awareness of the need to receive the HPV vaccination?

How could young MSM be encouraged to take up the HPV vaccine?

Prompts

- Awareness campaigns through schools, GUM clinics, social media etc.

7. Close *(2-3 mins)*

That is the end of my questions. Before we finish is there anything I haven't covered today that you would like to add?

We would like you to read our interpretation of the focus group. This shall be done by us sending you an email summary of the group discussion. We would like you to let us know if you feel it is an accurate interpretation of what was discussed. If you would like to do this, please provide us with an email address. This will not be kept confidential, and only used for this purpose.

End audio recording

Thank participant(s) and answer questions.

For peer review only

For peer review only - http://bmjopen.bmj.com/site/about/guidelines.xhtml

No	Item	Guide questions/description	Response and/or page no.
**Domain 1: Research team and reflexivity**			
Personal Characteristics			
1.	Interviewer/facilitator	Which author/s conducted the interview or focus group?	CF conducted the focus groups – page 7
2.	Credentials	What were the researcher's credentials? *E.g. PhD, MD*	CF has a PhD
3.	Occupation	What was their occupation at the time of the study?	Research fellow
4.	Gender	Was the researcher male or female?	Female
5.	Experience and training	What experience or training did the researcher have?	CF had qualitative research experience through her PhD
Relationship with participants			
6.	Relationship established	Was a relationship established prior to study commencement?	CF arranged the focus groups with

For peer review only

No	Item	Guide questions/description	Response and/or page no.
			participants
7.	Participant knowledge of the interviewer	What did the participants know about the researcher? e.*g. personal goals, reasons for doing the research*	Participants were informed of the research projects objectives. The participants knew that a researcher from Queen's University Belfast was conducting the focus groups.
8.	Interviewer characteristics	What characteristics were reported about the interviewer/facilitator? e.g. *Bias, assumptions, reasons and interests in the research topic*	None
**Domain 2: study design**			
Theoretical framework			
9.	Methodological orientation and Theory	What methodological orientation was stated to underpin the study? *e.g. grounded theory, discourse analysis, ethnography, phenomenology, content analysis*	The research took an applied methodological orientation, aligning most closely with grounded theory, rather than a more

For peer review only

No	Item	Guide questions/description	Response and/or page no.
			formal approach.<br><br>Page 8
Participant selection			
10.	Sampling	How were participants selected? *e.g. purposive, convenience, consecutive, snowball*	Convenience and snowball<br><br>Page 7
11.	Method of approach	How were participants approached? e*.g. face-to-face, telephone, mail, email*	A range of approaches were used. Potential participants were provided with written information both face-to-face at community LGBTQ advocacy groups and the study was advertised online<br><br>Page 7
12.	Sample size	How many participants were in the study?	17
13.	Non-participation	How many people refused to participate or	1 person left the focus

For peer review only

No	Item	Guide questions/description	Response and/or page no.
		dropped out? Reasons?	group before it began due to time constraints.<br><br>Due to the nature of the recruitment method we do not know how many people received the participant information sheet but did not participate. No one refused to participate to the researcher.<br><br>Table 1
Setting			
14.	Setting of data collection	Where was the data collected? e.g. *home, clinic, workplace*	LGQTQ advocacy groups and a University Student's Union<br><br>Page 7
15.	Presence of non-participants	Was anyone else present besides the participants and researchers?	No

For peer review only

No	Item	Guide questions/description	Response and/or page no.
16.	Description of sample	What are the important characteristics of the sample? *e.g. demographic data, date*	Table 1
Data collection			
17.	Interview guide	Were questions, prompts, guides provided by the authors? Was it pilot tested?	The interview topic guide is described on page 7.<br><br>The topic guide was not pilot tested but the questions were applied flexibility in a semi-structured manner.<br><br>Supplementary material B.
18.	Repeat interviews	Were repeat interviews carried out? If yes, how many?	No
19.	Audio/visual recording	Did the research use audio or visual recording to collect the data?	Yes, encrypted digital audio recording equipment.<br><br>Page 8
20.	Field notes	Were field notes made during and/or after the	Yes, field notes were

For peer review only

No	Item	Guide questions/description	Response and/or page no.
		interview or focus group?	made following each focus group.
21.	Duration	What was the duration of the interviews or focus group?	The focus group length averaged 44.34 minutes (range=40.4-50.4)<br><br>Page 9
22.	Data saturation	Was data saturation discussed?	Yes<br><br>Page 7 and 14
23.	Transcripts returned	Were transcripts returned to participants for comment and/or correction?	No
**Domain 3: analysis and findings**z			
Data analysis			
24.	Number of data coders	How many data coders coded the data?	JK and CF independently coded the first transcript systematically, line-by-line, compared their coding and reached consensus

For peer review only

No	Item	Guide questions/description	Response and/or page no.
			Page 8
25.	Description of the coding tree	Did authors provide a description of the coding tree?	No
26.	Derivation of themes	Were themes identified in advance or derived from the data?	Derived from the data<br><br>Page 8
27.	Software	What software, if applicable, was used to manage the data?	QSR NVivo 10.0<br><br>Page 8
28.	Participant checking	Did participants provide feedback on the findings?	No
Reporting			
29.	Quotations presented	Were participant quotations presented to illustrate the themes / findings? Was each quotation identified? e.g. *participant number*	Yes, quotations are presented with unique identifiers
30.	Data and findings consistent	Was there consistency between the data presented and the findings?	Yes
31.	Clarity of major themes	Were major themes clearly presented in the findings?	Yes
32.	Clarity of minor	Is there a description of	Yes

For peer review only

No	Item	Guide questions/description	Response and/or page no.
	themes	diverse cases or discussion of minor themes?	

For peer review only
